# Membrane protein contact and structure prediction using co-evolution in conjunction with machine learning

**DOI:** 10.1371/journal.pone.0177866

**Published:** 2017-05-24

**Authors:** Pedro L. Teixeira, Jeff L. Mendenhall, Sten Heinze, Brian Weiner, Marcin J. Skwark, Jens Meiler

**Affiliations:** 1 Department of Biomedical Informatics, Vanderbilt University Medical Center, Nashville, Tennessee, United States of America; 2 Department of Chemistry, Center for Structural Biology, Vanderbilt University, Nashville Tennessee, United States of America; University of Michigan, UNITED STATES

## Abstract

*De novo* membrane protein structure prediction is limited to small proteins due to the conformational search space quickly expanding with length. Long-range contacts (24+ amino acid separation)–residue positions distant in sequence, but in close proximity in the structure, are arguably the most effective way to restrict this conformational space. Inverse methods for co-evolutionary analysis predict a global set of position-pair couplings that best explain the observed amino acid co-occurrences, thus distinguishing between evolutionarily explained co-variances and these arising from spurious transitive effects. Here, we show that applying machine learning approaches and custom descriptors improves evolutionary contact prediction accuracy, resulting in improvement of average precision by 6 percentage points for the top 1L non-local contacts. Further, we demonstrate that predicted contacts improve protein folding with BCL::Fold. The mean RMSD100 metric for the top 10 models folded was reduced by an average of 2 Å for a benchmark of 25 membrane proteins.

## Introduction

Determining membrane protein (MP) structures experimentally is difficult as they are often too large for nuclear magnetic resonance experiments and remain difficult to crystallize [1]. Only about 2% of reported structures are MPs [2] and only around 100 distinct folds of integral helical MPs with more than one transmembrane (TM) span [3] are represented in the Protein Data Bank (PDB) [2, 4, 5]. At the same time, understanding MP structure is critical, as it is estimated that MPs comprise 15–39% [6] of the human proteome. They are also particularly important players in cell-environment interactions, i.e. environment sensing receptors, transporters, and channels. Accordingly, approximately half of current therapeutics target MPs[7]. Given the relatively small number of experimental structures, structure-based drug discovery is underdeveloped for MPs. However, as better models for MPs become available structure-based drug discovery will offer an avenue for developing new and more targeted pharmaceuticals [7]. Computational methods that can assist in *de novo* MP structure determination are therefore of increasing relevance.

One avenue for tackling the experimental difficulties in membrane protein structure determination is *de novo* protein folding. This approach, while theoretically sound, suffers from insurmountable computational complexity due to exponential dimensionality of the conformational space to be searched. The vastness of the search space can be significantly reduced by introduction of conformational restraints (hereinafter “restraints”), which often take form of upper limits on inter-atom distances (“contacts”). While the definition of a contact may differ between different works, the most commonly used criterion [8] is an amino acid pair with Cβ carbons within 8Å or less (Cα is used in the case of glycine). For the purpose of this work, we will neglect local, short-range contacts (with separation of at most 5 amino acids in the primary sequence), as they are not conducive for successful protein folding, focusing rather on non-local contacts, in short (6–11 amino acids), medium (12–23 amino acids), and long range contacts (24+ amino acids separation) [9, 10]. For sake of clarity, we will designate all contacts with separation of at least 12 amino acids as “long-range contacts”, instead of “medium- and long-range contacts”.

Until recently, contact prediction in proteins was of limited use for guiding protein folding, with leading methods being able to predict approximately 1 in 5 contacts correctly within a set of L/5 predictions (where L is the length of protein sequence) [9, 11–13]. Subsequent advances employing multiple layered artificial neural networks (ANNs) were able to improve this number to 30% [12]. Support vector machines have also been used to predict TM contacts[14]. Our previous work in the Bio Chemical Library (BCL) suite, which uses only sequence information, yields positive predictive values no higher than 42%[15]. Recent improvements in contact prediction [16–18] successfully applied the statistical methods used for solving inverse problems in statistical mechanics to the contact prediction problem. These methods rely on the idea of global statistical inference using co-evolutionary information encoded in the multiple sequence alignments (MSA) of evolutionarily related (homologous) proteins to assess the statistical couplings between individual positions in the protein. In contrast to older methodology, these methods, collectively referred to as Direct Coupling Analysis (DCA)[17–19], model the entire data set at once and not only independent pairs of residues, assuming the evolutionary interaction model is an instance of a high-dimensional Ising model on a fully connected graph. In so doing, they reduce the systematic errors of direct co-evolutionary methods (such as Mutual Information), where high correlation between two residues is an indirect consequence of both being highly correlated to a third residue. This in turn results in a significant boost in predictive power of the co-evolutionary methods, which in some case are able to obtain nearly perfect predictive performance within the set of top L predictions.

DCA-like methods, while successfully applied to contact prediction problem, do not actually predict contacting amino acids, but rather indicate the pairs of amino acids that are under evolutionary pressure to co-evolve[17, 18, 20]. While such a pressure is likely to originate due to spatial proximity, it may also be a result of functional constraints, oligomeric state or conformational flexibility of a protein. Furthermore, the inferred couplings are specific to a particular MSA and may differ substantially upon the change of the homology threshold for inclusion of a sequence in the MSA.

Here we combine mean field DCA (mfDCA) [17] with a variety of custom descriptors as an input to a machine learning approach. The resulting method surpasses the predictive capabilities of mfDCA (including subsequent filtering steps advised by method's authors) in terms of precision by 6% on average, up to 17% (for aquaporin 0, PDB id:1YMGA). While this improvement has been measured for the top L long-range contacts (i.e. pairs of separation of at least 12 amino acids) using the ANN approach, we show that the improvement is relatively agnostic to the machine learning method used, by replicating the results using decision trees (DTs). Finally, we demonstrate, that these predicted contacts improve MP folding with BCL::Fold on average by 2Å in comparison to the same protocol without contact restraints.

## Materials and methods

### Membrane protein benchmark data set and alignment preparation

This work focuses on the contact and structure prediction of a set of 25 diverse α-helical membrane proteins of known structure, having more than four transmembrane helices. The data set has been chosen, so that for each target one can identify sufficiently many homologs (more than 1000) with satisfactory coverage to warrant success with coupling inference. Site-coverage (Cov) is the percent of the target sequence sites that map onto the final MSA after one removes columns with a large number of gaps. Based on prior work [17], a threshold of 30% gaps was used for this analysis. These 25 membrane proteins come from 23 Pfam families [21], contain a maximum of 15 helices, and have a maximum initial target length of 572 [22].

It is not evident what makes for the most suitable multiple sequence alignment for coupling inference. Most of the work published so far either chooses the alignment generation parameters arbitrarily [18] uses external, oftentimes intuition-based approaches [22] or samples multiple alternative alignments [20, 23, 24]. We created alignments for each protein using the HMMER3 software package [25] at the same E-values—1E-3, 1E-5, 1E-10, 1E-15, 1E-20, 1E-30, and 1E-40.

As many of the DCA-like methods are sensitive to the alignments containing large unaligned stretches (gaps), we have pruned the input alignments to discard the sequences containing more than 30% of gaps. The statistics of filtered and unfiltered MSA are included in Table 1. Filtered MSA are significantly smaller—on average nearly 16 fold so. Maximum, minimum, and average unfiltered MSA sizes are 65525, 1503, and 17865 respectively. The maximum, minimum, and average filtered MSA sizes are 1745, 684, and 1142, respectively. Coverage increases nearly 16% from approximately 76% coverage in unfiltered to 88% in filtered MSA.

**Table 1:** The 25 membrane proteins used as the benchmark set.

Protein	Initial Sequence				Filtered			Unfiltered		
Name	PDBID	L	Opt. E_val_	TM_helix_	M_align_	M_eff_	Cov	M_align_	M_eff_	Cov
ADIC_SALTY	3NCYA	422	1.00E-20	12	1215	1205	0.891	16975	5560	0.884
ADRB2_HUMAN	2RH1A	442	1.00E-20	8	818	451	0.652	22822	5228	0.559
ADT1_BOVIN	1OKCA	292	1.00E-40	6	1068	1043	0.890	8631	3516	0.890
AMTB_ECOLI	1XQFA	362	1.00E-05	11	1048	1021	0.961	4051	1416	0.925
AQP4_HUMAN	3GD8A	223	1.00E-10	7	1073	1062	0.964	5400	1933	0.955
BTUC_ECOLI	1L7VA	324	1.00E-10	10	1049	1045	0.914	9399	4125	0.910
C3NQD8_VIBCJ	3MKTA	460	1.00E-20	12	1072	1068	0.917	11067	5591	0.913
C6E9S6_ECOBD	3RKON	473	1.00E-10	14	1745	1722	0.831	59616	5932	0.588
COX1_BOVIN	1OCCA	514	1.00E-40	12	1157	754	0.979	47394	1289	0.089
COX3_BOVIN	1OCCC	261	1.00E-03	6	684	521	0.958	9105	1444	0.709
CYB_BOVIN	1PP9C	379	1.00E-03	8	1069	581	0.921	49258	855	0.272
FIEF_ECOLI	3H90A	283	1.00E-05	6	1050	1039	0.968	7610	3473	0.933
GLPG_ECOLI	3B45A	180	1.00E-05	6	1092	1073	0.867	4625	2323	0.739
GLPT_ECOLI	1PW4A	434	1.00E-30	12	1611	1604	0.878	25199	10789	0.882
METI_ECOLI	3DHWA	203	1.00E-15	5	1086	1065	0.877	13418	4788	0.877
MIP_BOVIN	1YMGA	233	1.00E-10	7	1032	1010	0.901	5431	1937	0.897
MSBA_SALTY	3B60A	572	1.00E-03	6	1576	1568	0.881	65525	29777	0.388
O67854_AQUAE	2A65A	510	1.00E-03	12	1135	1043	0.825	4351	1657	0.818
OPSD_BOVIN	1HZXA	340	1.00E-20	7	1165	1151	0.803	40460	8873	0.782
Q87TN7_VIBPA	3PJZA	468	1.00E-10	12	1019	923	0.793	3340	1587	0.791
Q8EKT7_SHEON	2XUTA	456	1.00E-10	14	1055	1040	0.706	8196	2983	0.697
Q9K0A9_NEIMB	3ZUXA	308	1.00E-10	10	1024	1005	0.899	3928	1549	0.903
SGLT_VIBPA	2XQ2A	538	1.00E-05	15	1515	1380	0.820	8075	3177	0.784
TEHA_HAEIN	3M71A	306	1.00E-03	10	822	646	0.971	1503	735	0.948
URAA_ECOLI	3QE7A	407	1.00E-03	14	1371	1355	0.818	11244	3384	0.747
Statistics										
Mean		376	2.42E-04	9.68	1142	1055	0.875	17865	4557	0.755
Standard Deviation		109	4.26E-04	3.07	244	309	0.080	18581	5691	0.218
Maximum		572	1.00E-03	15	1745	1722	0.979	65525	29777	0.955
Minimum		180	1.00E-40	5	684	451	0.652	1503	735	0.089

Protein names and Protein Data Bank IDs (PDBID) are accompanied by the length of the initial target sequence L, the optimal E-value used to generate the alignment (Opt. Eval) [22], the number of transmembrane helices predicted with SPOCTOPUS (TM_helix_), in addition to the number of sequences in the created alignments (M_align_), the effective number of alignment sequences, which takes into consideration sequence diversity (M_eff_), and the percent coverage of the initial sequence by the final alignment with columns containing over 30% gaps removed (Cov). The MSA-related data is included for both filtered and unfiltered datasets.

### Coupling inference

In the interest of direct comparability with prior work, we have used the mean field DCA method employed by Morcos et al [17], which has been instrumental to successful folding approaches, such as EVfold [22, 26]. Therefore, for each of the alignments, we have re-weighted the sequences that share more than 80% identical amino acids, in the interest of reducing the bias of large homologous clusters on the inference process. Then we use the mean field principles to obtain the coupling matrix *D* for individual position pairs, which in this instance (naïve mean field DCA) reduces to computing an inverse of covariance matrix C^-1^.

Due to insufficient sampling of the sequence space, not all amino acid type pairs will be represented, therefore the covariance matrix is highly likely to be singular (non-invertible). In order to avoid this situation, Morcos et al. augment the covariance matrix with a high dose of pseudocounts (0.8 of the resulting weighted sum) derived from the known sequence database statistics.

The entries in the resultant coupling matrix *D* are not directly comparable between different positions in the protein. Therefore, in order to enable ranking of position pairs, we compute a Mutual Information-like score involving the individual positions in the matrix *D* (and their sums as proxies for single-site values), producing the scoring matrix *S*. Finally, the computed scores were mapped back to the original sequence coordinates (before gap-column removal) using the auxiliary script from HHsuite [27].

### Membrane protein topology prediction

Global methods for coupling inference, especially based on the mean-field approach, when applied to contact prediction suffer from systematic overprediction for evolutionarily coupled, but spatially distant sites. One of such overpredictions involves membrane protein sites located at the interface between membrane and aqueous environment.

In order to alleviate these and related issues, as well as introduce, what we believe to be, crucially important piece of prior data, we have incorporated the information on the predicted membrane topology and topography into the contact prediction process. To do so, we have used SPOCTOPUS [28], a two-track membrane protein topology predictor, that incorporates a signal peptide pre-filter. None of the protein sequences in our data set included signal peptide, thus rendering the method we used functionally identical to OCTOPUS [29]. To further enhance the predictive performance, we moved beyond three-state predictions of (SP)OCTOPUS to a numerical indicator of each amino acids membrane location. Positions predicted to be on the inner-membrane as part of a loop are assigned a value of zero (consistent with a SPOCTOPUS prediction of “*i*”). Positions predicted to be outside of the membrane as part of a coil are assigned a value of one (consistent with a SPOCTOPUS prediction of “*o*”). Transmembrane helices are predicted as “*M*” in SPOCTOPUS and their subsequent encoding is determined by the position within the helix (from inner to outer membrane) and normalized based on the helix size, which in case of (SP)OCTOPUS is always 21. Therefore, the amino acid of the transmembrane span closest to the outside of the membrane will receive value of 1/22, second 2/22 and so on, until the one closest to the inside of the membrane receives value 21/22, differentiating it from the subsequent outer-membrane loop amino acid. We treated the few short re-entrant helices encountered as inner or outer-membrane loops.

### Feature generation

**Fig 1:**
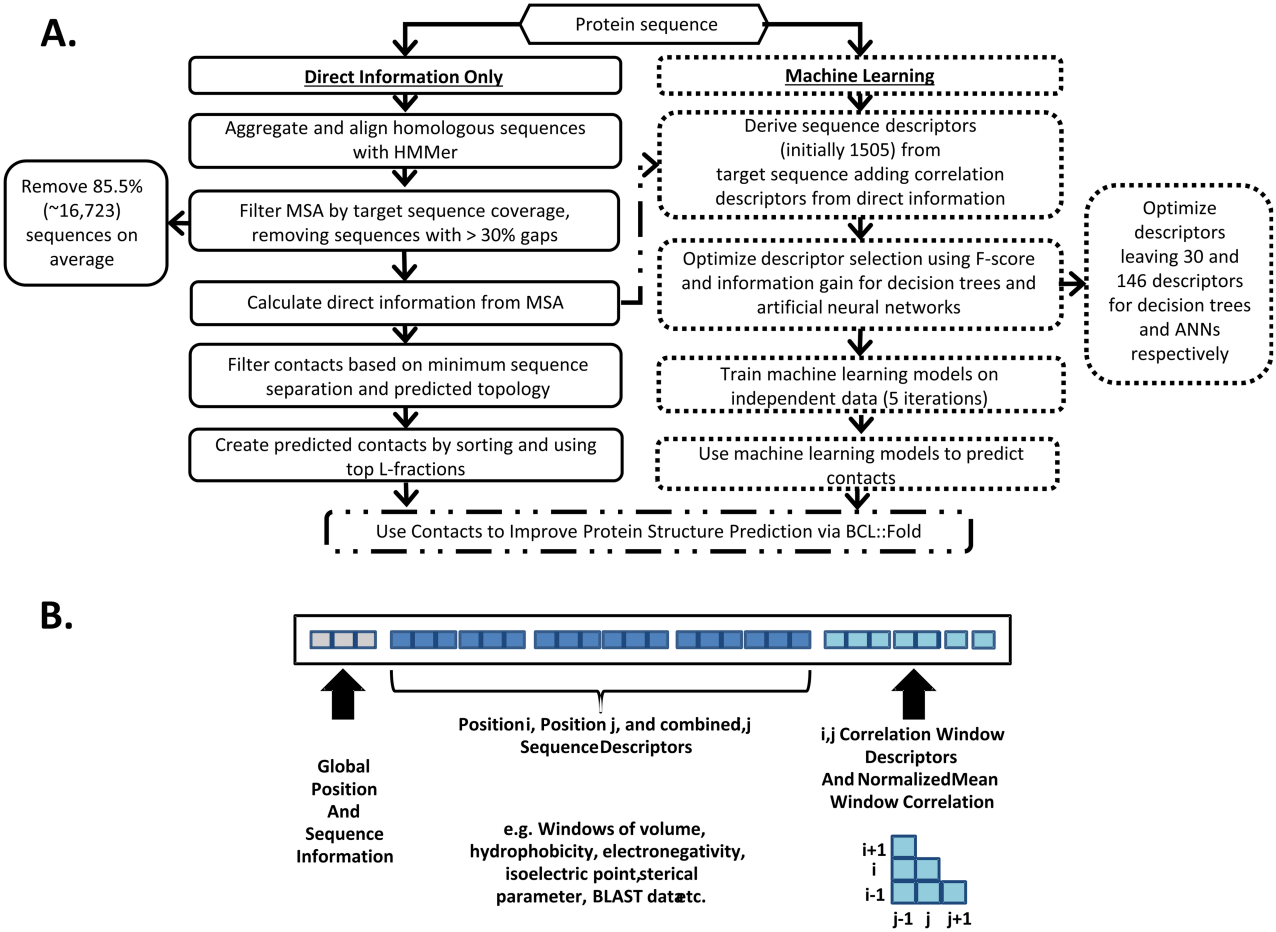
Contact prediction flowchart and diagram of resulting descriptor categories used for machine learning. A.) Flowchart overview for producing contacts—divided into the DI only method (solid black outline, left) and the all-inclusive machine learning sequences (dotted black outline, right). Both result in sets of the top L-fractions of predicted contacts, which were then used in combination with BCL::Fold to predict the structures of the 25 membrane proteins in the benchmark set. B.) Descriptor vectors include three categories: global, sequence information, and correlation descriptors. Global descriptors include sequence position for sites i and j, the separation from i to j, and the number of amino acids in the sequence. Sequence information descriptors include windows of biochemical properties surrounding sites i and j such as volume, hydrophobicity, sterical parameter, polarizability, isoelectric point, and BLAST profiles. The probability of each SSE state helix/strand/coil (by JUFO9D) as well as membrane/transition/solution state are also included. Finally, correlation information includes the symmetric matrix around sites i and j, all unique pairwise combinations from *i ± half_window_size* with *j ± half_window_size*, the mean, max, and normalized mean of this window, and the overall mean sequence correlation.

It is not obvious what additional prior information may assist the contact prediction for membrane proteins. Therefore, we have explored 1,505 additional descriptors (features) for training the machine learning approaches. A schematic depiction of a descriptor input vector is provided in Fig 1 panel B. S1 Table lists all the descriptor categories considered.

The most basic category includes global descriptors, that is amino acid positions for sites *i* and *j*–indexed starting from one, the separation between *i* and *j* (i.e. *|i-j|*), and total sequence length.

The inferred co-evolution couplings, which constitute a major improvement of this method over the previous iterations, were included both in a direct and aggregate way. Coupling inference using alignments at different homology thresholds tends to reveal slightly different information about protein's evolutionary history [23], therefore we have used couplings derived from both filtered and unfiltered alignments, at “optimal” set of E-values, as well as from all E-values. For each of the coupling sets, we included the information on the individual strengths of evolutionary information by using a nine residue window around each of the amino acids in question. The window size has been selected to enable capturing the information on two turns of alpha helices, which are predominant building blocks of MPs. In addition to raw coupling strengths, we have also incorporated the maximum, mean, standard deviation, normalized mean (see S1 File) and sum, both window-wise and protein-wise.

Sequence information descriptors comprise windows of biochemical properties of amino acids surrounding sites *i* and *j*. These include volume, hydrophobicity, steric parameter, polarizability, isoelectric point, and the Basic Local Alignment Search Tool (BLAST)-derived PSSM (sequence profile) for each site in the window pair. These sequence descriptors together with global descriptors were used previously in the BCL to predict long-range contacts Descriptor set includes also the statistics on the multiple sequence alignments used for the coupling inference, that is the length of the aligned target sequence including gaps, the depth of the alignments (raw number of non-identical sequences) and the effective depths of the alignments (M_eff_ S1 File) which allows to account for sequence redundancy, as well as target sequence coverage for the given MSA.

For prediction of a secondary structure element (SSE) propensity (helix/strand/coil), as well as the membrane state (membrane/transition/solution) for each amino acid position, we used JUFO9D [30]. It is a newer, internally developed multitrack ANN algorithm. Each individual ANN is trained on certain SSE type interactions, to improve accuracy. The SSE assignments for both sites were supplemented with SSE index difference, a descriptor indicating how many SSEs are between these amino acids (if both amino acids are in the same SSE, the descriptor equals 0, if they are in neighboring SSEs– 1 etc.). Finally, we have supplemented this information with position-aware descriptors. They are related to the size of SSEs containing amino acids in question and contain their distance from the center of respective SSEs. In particular, we have encoded the vertical membrane position of considered amino predicted by using this approach.

### Feature selection and learning the statistical model

In order to avoid overfitting to the training data and improve robustness of the methods, we have iteratively pruned the set of descriptors in order to find a minimal set of descriptors, that captures the information essential for MP contact prediction. The pruning was achieved by ranking descriptors by F_1_-score (harmonic mean of precision and recall, S1 File) and information gain (the difference of the information entropy after partitioning the set using the given descriptors, Kullback-Leibler divergence–S1 File).

The statistical models trained for the purpose of this work were obtained in a two-step process. In order to ascertain the most suitable machine learning approach, we have trained both ANNs and DTs. For both approaches, we have first performed initial training with all the potential descriptors. To conduct the training, we have performed five-fold cross-validation, by splitting the proteins randomly between training, monitoring and independent (testing) set, of 15, 5 and 5 proteins respectively. No data from the independent set was ever used to optimize parameters or train the machine learning model. We were thus able to optimize the method using entirety of our protein set, while at the same time avoiding cross-contamination, as no data points appeared in more than one of the data set simultaneously. As protein pairs 3GD8A/1YMGA and 2RH1A/1HZXA share non-negligible sequence similarity, they have been always included together in the same data set.

Initial stages of training used root mean square error (RMSE) as the optimization criterion. As DTs are prone to overfitting, we have forbidden splitting the nodes (making tree deeper), if the size of the node was less than 20 data points. For ANN learning, we have established the **α** (learning momentum) and **η** (gradient descent contribution) parameters by grid search in cross-validation regime. The values that provided the best results were **η** equal to 0.000017 and **α** equal to zero.

For the DT-based approach, the first round of optimization has used F_1_-score (harmonic mean of precision and recall) and information gain (Kullback-Leibler divergence), which has demonstrated that only 210 of the descriptors considered contributed significantly to the predictive performance.

This set of descriptors have been then iteratively pruned in respect to the measured input sensitivity [31, 32]. We then iteratively rescored the top remaining descriptors (starting from this set of 210) with input sensitivity using the best run from the previous stage. The best threshold was set as the new top descriptor threshold. To decrease the likelihood of removing useful descriptors, no more than half were removed at each stage. Thus, we subsequently examined the top 160, 130, and 70 descriptors. In the third iteration (top 70 descriptors), approximately top 30 descriptors resulted in the best performance. We used the average of the enrichment as the objective function, where enrichment is the fold increase in positives. We evaluated each set of models generated by calculating the integral of the precision over the range 0.01% to 0.55% of the fraction predicted positive. This range closely captures the contacts predicted for the top L predictions across all proteins while avoiding the noise present below 0.01%. The small number of data points results in drastic changes from small perturbations in overall predictions below 0.01%.

In case of ANN based approach, we use the method for descriptor selection that has already been introduced and tested in BCL. We approximate the derivative of the effect of each feature column on the results, as measured by the weights of trained ANN. This derivative is then used in two ways to score descriptors: consistency of effect (i.e. does the derivative’s sign remain constant across all models in the cross validation ensemble, thus consistently increaseing/decreasing the emitted likelihood of contact) and the mean of the square of derivative. To compute the latter metric, we rescale each derivative between 0–0.5, sum the scaled values and square their values. The underlying rationale is that insignificant, noisy features will have low weights, roughly equal to zero. However, it is possible for the non-noise features to have a non-linear relationship to the output, in which the mean weight (and the mean derivative) would be close to zero, but most of the weights would not, which is the situation this metric aims to detect.

After selecting the optimal set of parameters and descriptors, we conducted final training, using the same procedure, but substituting average enrichment in place of RMSE.

### BCL::Fold protein structure predictions

BCL::Fold creates each model through a Monte Carlo optimization with two stages. It begins model assembly by either placing, removing ir performing large SSE-based moves. The second stage focuses on the refinement of the generated models and utilizes small amplitude SSE translations and rotations to arrive at a final model. After each SSE move, models are scored using knowledge-based potentials that examine MP topology, environment prediction accuracy, SSE alignment, radius of gyration, amino acid environment, contact order, amino acid clashes, and a loop score [33–35].

The knowledge-based potential, BCL::Fold may be augmented with prior knowledge, in case of this work—contact information. As predicted contacts are imperfect, additional terms should encourage meeting the restraints, but not punish large violations. To do so, we have used a smooth step function with two parameters or “threshold values”. If restrained amino acids' carbon alpha atoms are not further than 8Å (upper bound of the contact range), restraint function confers a -1 bonus to the score. The other parameter is the width of transition region, 12Å, a maximal distance above which restraint does not affect the score anymore. The values of restraint function between these two thresholds are dictated by a smooth, monotonic function, characterized by a zero derivative at threshold points—a the negative of the sine function in range [0, pi/2] where the output decreases from 0 to the maximal penalty of -1. The penalty plateaus as separation approaches 20Å, thus limiting the influence of sites well beyond the contact range.

Here we used the mean of the predicted contact propensities output by each of the five cross-validated classifiers (both for DT and ANN based approaches). For each protein, we have restrained the top *L* highest scoring amino acid pairs to be in contact and ran the folding protocol independently and in parallel, generating 1,000 models for each protein and machine learning approach, producing 50,000 total models.

As the main goal of this work is to demonstrate the effect of contact prediction on protein folding, we have used top 10 best models by RMSD100 to the native structure. Performing a second iteration of folding did not significantly alter results.

## Results and discussion

### Topology filtering DCA-derived contacts improves prediction precision

While evolutionary coupling inference has been shown to be successfully applicable to protein contact prediction [17, 18, 23, 24, 26, 36], not every highly coupled amino acid pair corresponds to close proximity in the native structure. This is due to three confounding factors in evolutionary coupling inference: (i) method limitations, (ii) missing data, and (iii) non-spatial coupling causes. The statistical methods used for coupling inference make certain assumptions about the model of protein evolution, including the assertion that co-evolution analysis can be reduced to a two-site inverse problem. While not unreasonable, there is no evidence that certain evolutionary effects could not be modelled more aptly as n-body interactions. To do so would require substantially more sequence data than currently available. The other assertion that these methods make is that the input multiple sequence alignment captures the evolutionary history of a protein accurately. It is a fact, that multiple sequence alignments available currently are not uniformly samples from evolutionary history and consequently lack the ancestral sequences, as well as potentially include sequences of proteins that are not homologous to the protein of interest. Finally, even given the most accurate method and infinite data, there may be other sources of evolutionary couplings, including but not limited to: homo-oligomers with evolutionary pressures across protein interfaces that confound intra-protein contact prediction, selection pressures across proteins with multiple receptor/signaling domains that are evolutionarily related but not in physical proximity and other functional constraints.

As demonstrated before [20, 24], use of prior knowledge on protein structure substantially improves the contact prediction accuracy. The initial approaches to using evolutionary couplings to solve protein folding problem filtered away the pairs that were implausible to form contact, which led to a substantial increase in expected folding accuracy [22]. For example, two sites predicted to be on either end of a transmembrane α-helix are very unlikely to be in contact. However, such a pair may yield a high coupling value if they are part of a receptor-signaling pathway, which constrain their evolution similar to the functional roles that rely on the physical proximity.

**Fig 2:**
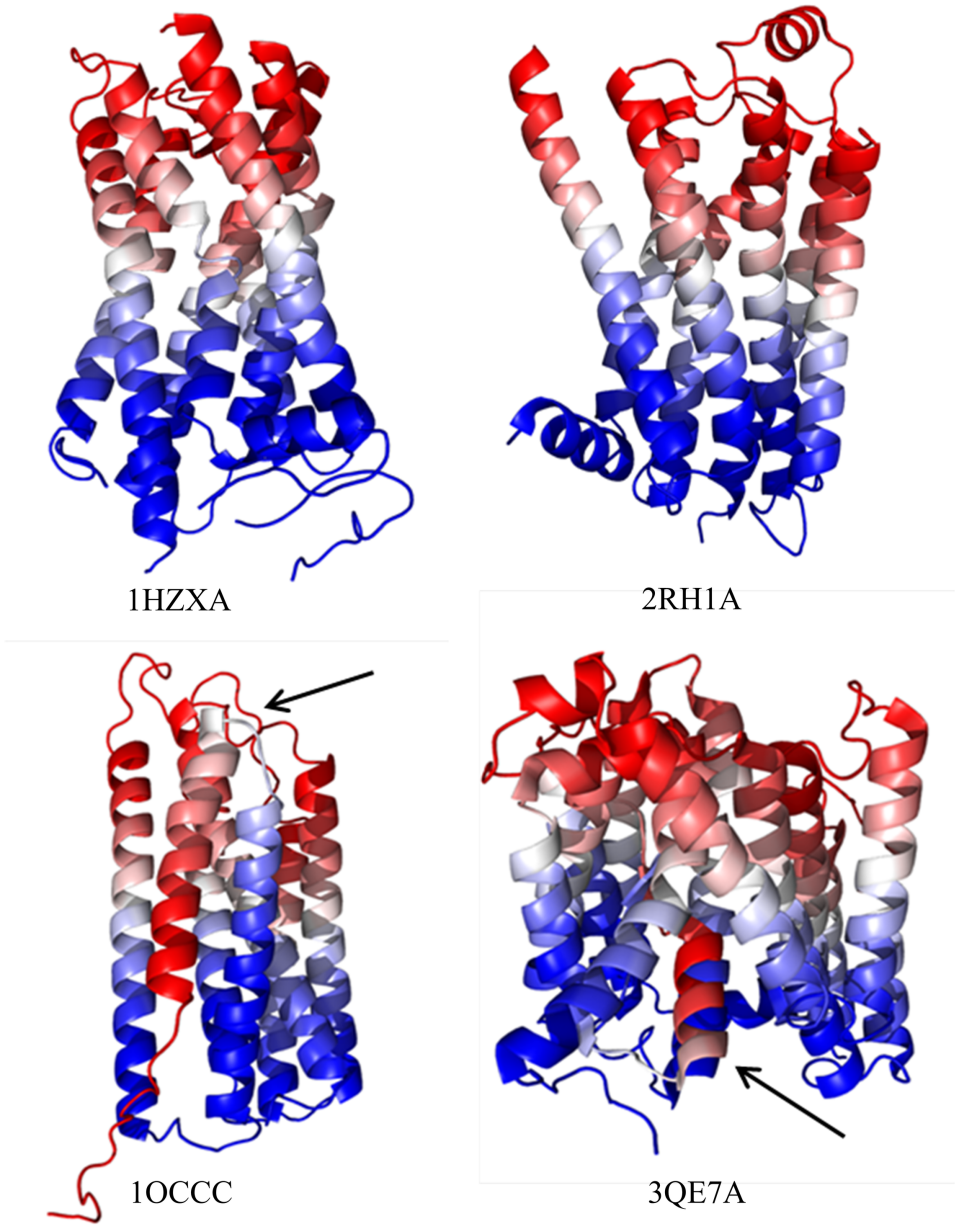
Visualization of the predicted transmembrane position descriptor. We used SPOCTOPUS to predict topology and then assigned each amino acid within all proteins a 0 for inner membrane (blue), 1 for outer membrane (red), and a value between 0 and 1 based on the distance along the predicted transmembrane helix normalized by the size of the containing helix. Above are 1HZXA, 2RH1A, 1OCCC, and 3QE7A (top left to right, bottom left to right). The vast majority (23 of the 25 benchmark proteins) are similar to the top two examples—well defined and aligned gradients across the structure from inner to outer membrane portions (blue to red). The only two proteins with significant errors are on the bottom (1OCCC and 3QE7A). One can see in 1OCCC that the foremost helices do not align to the expected gradient due to their inaccurate prediction as a single unbroken helix. A similar error exists in 3QE7A where a small portion is incorrect due to a missing helix break prediction.

The transmembrane position descriptor (see Methods), that we have developed, attempts to encode this observation. Fig 2 demonstrates that this descriptor results in a very consistent gradient along a blue-white-red (0–1) spectrum (inner to outer membrane) with similar colors in close vertical proximity. The mostly perpendicular orientation of the α-helices within MPs simplifies vertical separation prediction. Amino acids with different colors are unlikely to be in spatial contact.

For the original mfDCA filtered and processed method to which we compare, we used a threshold of 0.35 predicted vertical separation units, above which couplings are discarded as implausible. This threshold appears to be optimal in regard to the final accuracy for all L-fractions including ranges ideal for contact-driven protein structure prediction.

### Aggregate descriptors improve contact prediction with decision trees and artificial neural networks

Out of the 1,505 features assessed for the potential to improve protein contact prediction, by far the most promising were the aggregated, coupling-related descriptors. They ranked high in respect to F-score, information gain [37], and input sensitivity [31, 32, 38]. These included the normalized mean, window maximum and mean direct information (DI), and maximum and mean DI across larger sets of all calculated MSA, using various e-values, both with and without filtering. As demonstrated before [20, 23], the use of summary statistics combined with multiple sources of co-evolutionary information allows for separating spatially-related couplings from the ones arising due to the confounding factors introduced by suboptimal input alignments.

The descriptor which proved particularly useful is the normalized mean coupling value. It reduces the impact of discrepancies in coupling values between proteins by dividing the mean coupling score for a square sub-matrix by the mean coupling score across the entire protein sequence (S1 File). This descriptor implicitly captures a phenomenon employed by other methods, that is that pairs with high couplings corresponding to spatial proximity tend to have neighbors that have high coupling values as well, while this regularity is usually not present for the spurious ones. The size of a window is a compromise between capturing sufficient amount of information and generalizability. A window size of nine was chosen to capture two complete windings of an α-helix. Shorter and longer window sizes were tested but yielded worse results (data not shown).

In the final optimized set of 30 descriptors that we used for DTs (see Methods and S2 Table), 18 involved coupling values, out of which 12 were aggregate metrics. Moreover, 9 of these features are estimated to be of greater importance for prediction performance than the first “simple” co-evolution descriptor. The input sensitivity of the highest ranked aggregate descriptor is also almost 30 fold higher than that of descriptor based on DI alone.

The highest ranked descriptor is the correlation window maximum using filtered MSA created with the optimal E-values. Coincidentally, this set of DI values also performs best for naïve DI contact prediction. The second highest by input sensitivity is the sequence separation, which is partially due to positions closer in sequence being more likely to be in contact, but also captures the periodicity of certain contacts (e.g. helix-helix contacts, where contact between *i*,*j* implies lack of contact between *i±2*,*j±2*). The third highest-ranking descriptor is transmembrane separation predicted by the topology filter. The normalized window mean and maximum DI from the unfiltered optimized MSA are also ranked highly. Polarizability is the highest ranked traditional biochemical property descriptor but in this case it is an aggregated sequence mean and not just the polarizability for positions *i* and/or *j*.

**Fig 3:**
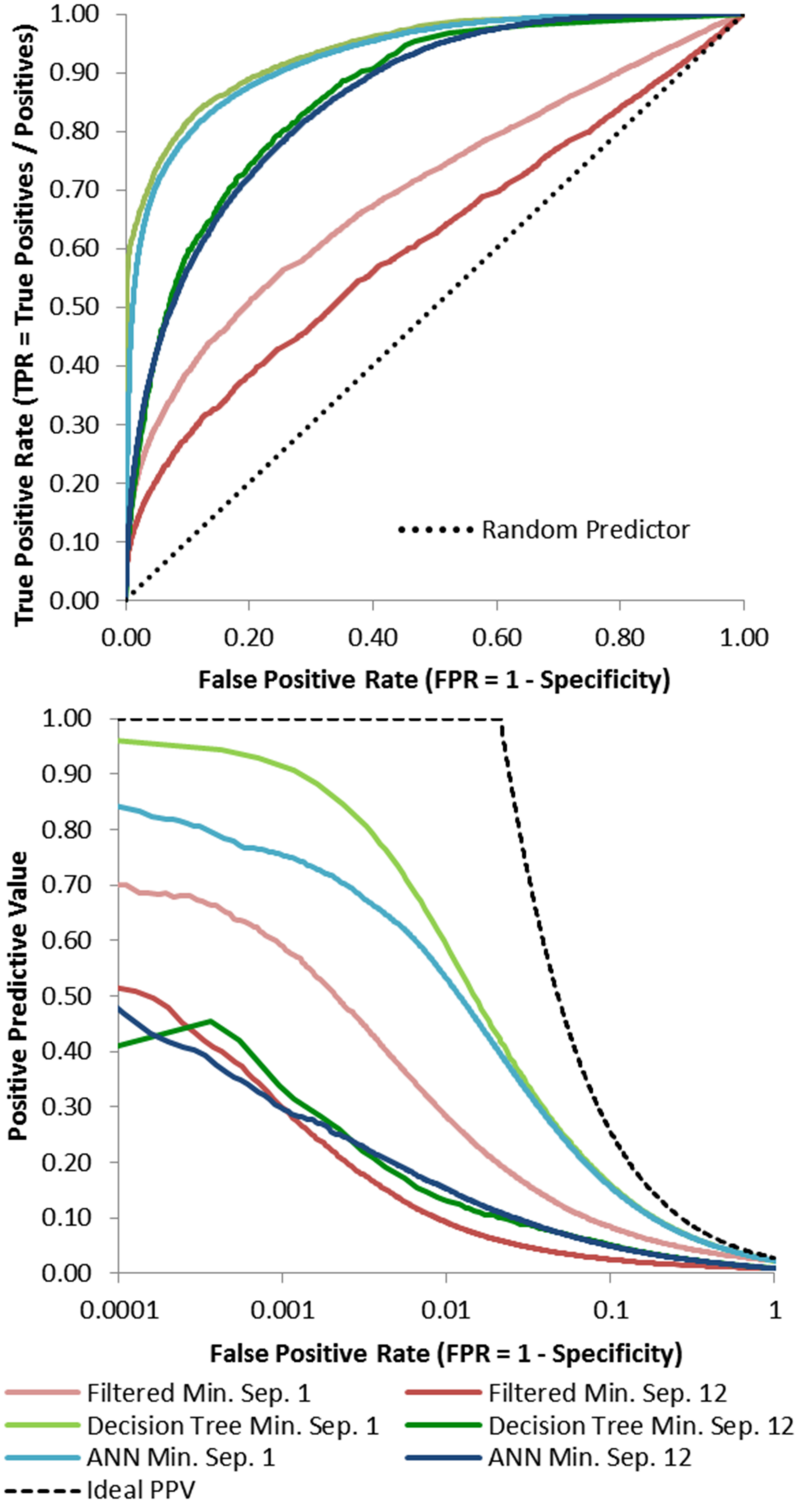
Best DT and ANN contact prediction ROC curve and logarithmic precision vs. fraction positive predicted (FPP) compared to naïve direct information with minimum separations of 1 and 12. ROC curves of the merged predictions averaged from five different training iterations for each protein. Also included are results from contact prediction based solely on naïve direct information (using the optimal filtered MSA) for comparison. The training, monitoring, and independents set included data from 15, 5, and 5 proteins respectively. The independent predictions are the ones presented above. AUC is approximately 0.700, 0.938, and 0.928 at a minimum separation of 1 for the filtered direct information, DT, and ANN methods respectively. For a minimum separation of 12 the AUC is approximately 0.611, 0.862, and 0.855 for the filtered DI, DT, and ANN methods respectively. Both methods significantly outperform naïve DI with a slight edge for DTs. The bottom panel contains a graph showing precision as the fraction predicted positive increases. The black line depicts ideal performance. Each curve includes the aggregated predicted contacts from five training iterations using DTs or ANNs. Models were trained using all contacts with a minimum separation of 1 and were tested on pairs with a minimum separation of 12. Each iteration uses 15, 5 and 5 proteins for the training, monitoring, and independent sets respectively. The integral of the precision from 0.01% to 0.55% is approximately 0.656, 1.921, and 0.865 at a minimum separation of 1 for direct information, DT, and ANN based methods respectively. At a minimum separation of 12 the integral is approximately 0.537, 0.469, and 0.549 for direct information, DT, and ANN based methods respectively. Greater precision initially and continuing out as FPP increases is better.

Fig 3 displays the receiver operating characteristic (ROC) curves for DTs and ANNs for predictions across the entire benchmark set in comparison to naïve DI using filtered MSA at minimum separations of twelve. We also include results for a sequence separation of one. Most notably, the AUC is higher for both DTs (0.862) and ANNs (0.855) in comparison to naïve DI (0.611). However, the goal is to predict a small fraction of all theoretically possible contacts with especially high accuracy. DTs and ANNs outperform naïve DI for the key false-positive-rate range of 0.001 to 0.1.

**Fig 4:**
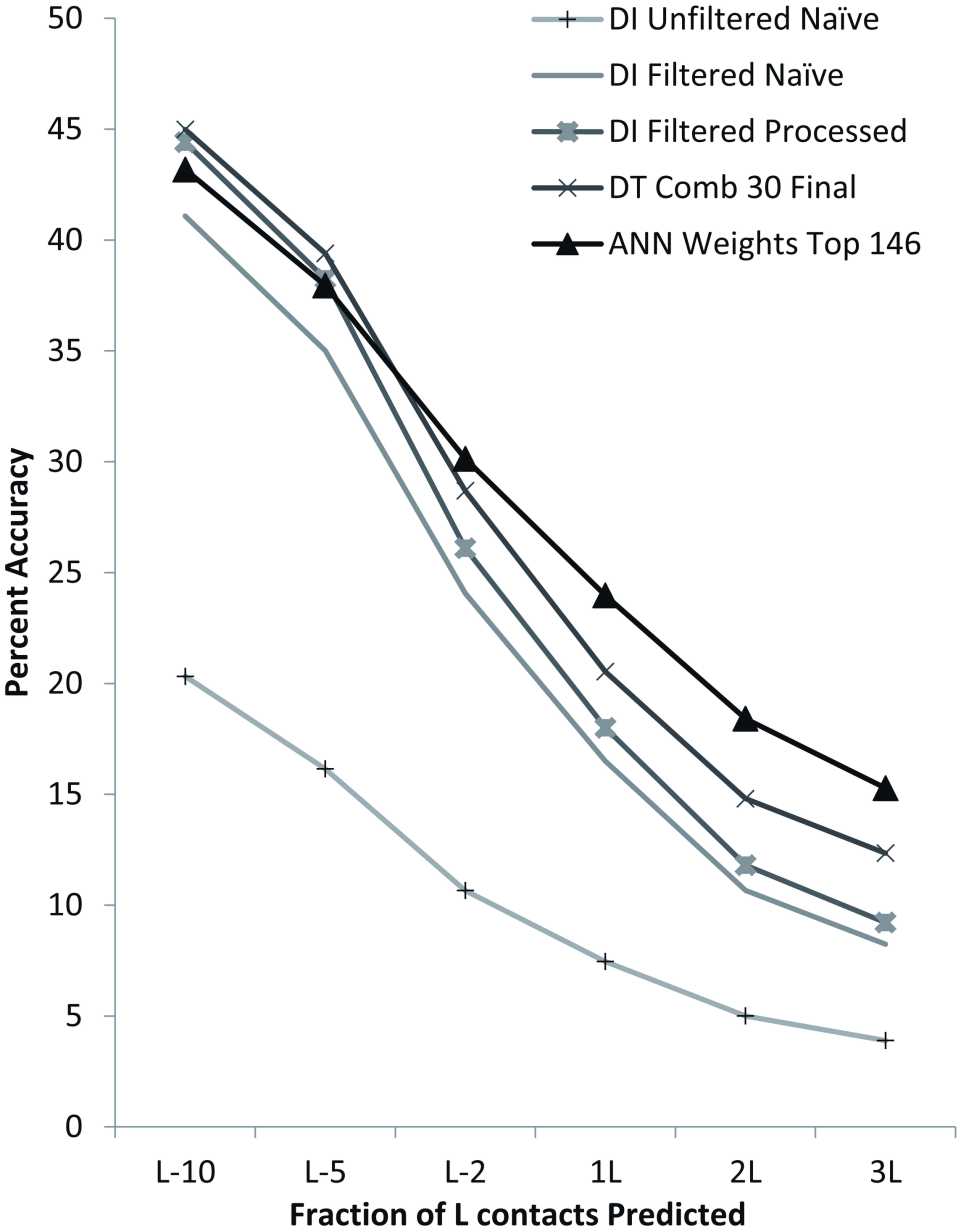
Accuracy comparison across best DT, ANN, naïve direct information, and processed direct information contact prediction for a minimum separation of 12. The graph above depicts the average accuracies of each method across the entire benchmark set for each of the top L fractions examined. Accuracy is significantly higher for DI contact predictions from filtered MSA in comparison to unfiltered and is further improved by processing (filtering based on predicted transmembrane topology). The processed method is second best for the top L/10 predictions (44.42%) only slightly lower than the best DTs (44.99%). DTs are also best for the top L/5 (39.40%). For L/2, 1L, 2L and 3L ANNs optimized using an analysis of weights between nodes produces the best results (30.14%, 23.97%, 18.41%, and 15.28% respectively). Thus, for all L-fractions, one or more commonly both machine learning methods have a higher average accuracy.

Fig 4 compares prediction accuracy at a minimum separation of twelve between naïve DI using filtered and unfiltered MSA, processed DI on filtered MSA, and the best DT and ANN sets across varying top L fractions. DTs outperform all DI-only methods. The ANNs outperform all methods, including DT-based methods, for L-fractions of L/2 and higher. The ANN’s advantage increases with larger fractions of L. Full results are given in S3 Table. The best accuracy for each PDB ID and L-fraction is marked in bold. ANNs have the highest accuracy at 3L and a minimum separation of twelve for 19 of the 25 benchmark proteins.

To put these results in context of currently established contact prediction methods, we have *calculated positive predictive values for* predictions with PSICOV[39], CCMpred [40] and FreeContact [41], using the same alignments as used by the methods we describe (S4 Table).

### Known contacts improve BCL::Fold structure predictions

Accurate contact restraints limit the fold search space thereby enabling more efficient conformational sampling. For methods such as BCL::Fold [34, 35], the smaller search space increases the likelihood of sampling native-like topologies. BCL::Fold is especially well suited to incorporating contact restraint information as the primary sequence is distilled down to SSEs with intervening loop regions removed during folding [42]. Thus, SSEs can move more easily to satisfy restraints. For the full-sequence single-chain models, reaching long-range restraints may be kinetically or energetically inaccessible, due to relatively large perturbations needed to overcome local minima. We can subsequently rebuild loops for the best scoring models, thus rendering fully-fledged models.

In order to illustrate the extent to which BCL::Fold may take advantage of contact restraints, we have attempted to recover the structures of proteins in our data set by folding them with a varying number of contact restraints derived from experimentally determined structures. As expected, increasingly large L-fractions of correct restraints improve results (S1 Fig). For the beta2-adrenergic G protein-coupled receptor (PDB: 2RH1A) the best model sampled improved from 4.5Å to 2.5Å. For aquaporin 4 (PDB: 3GD8A) and cytochrome C oxidase (PDB: 1OCCA) the effect is less dramatic but present. This shift to lower RMSD100 values across models persists across the benchmark set and represents the positive control.

### Predicted contacts improve BCL::Fold structure predictions

**Fig 5:**
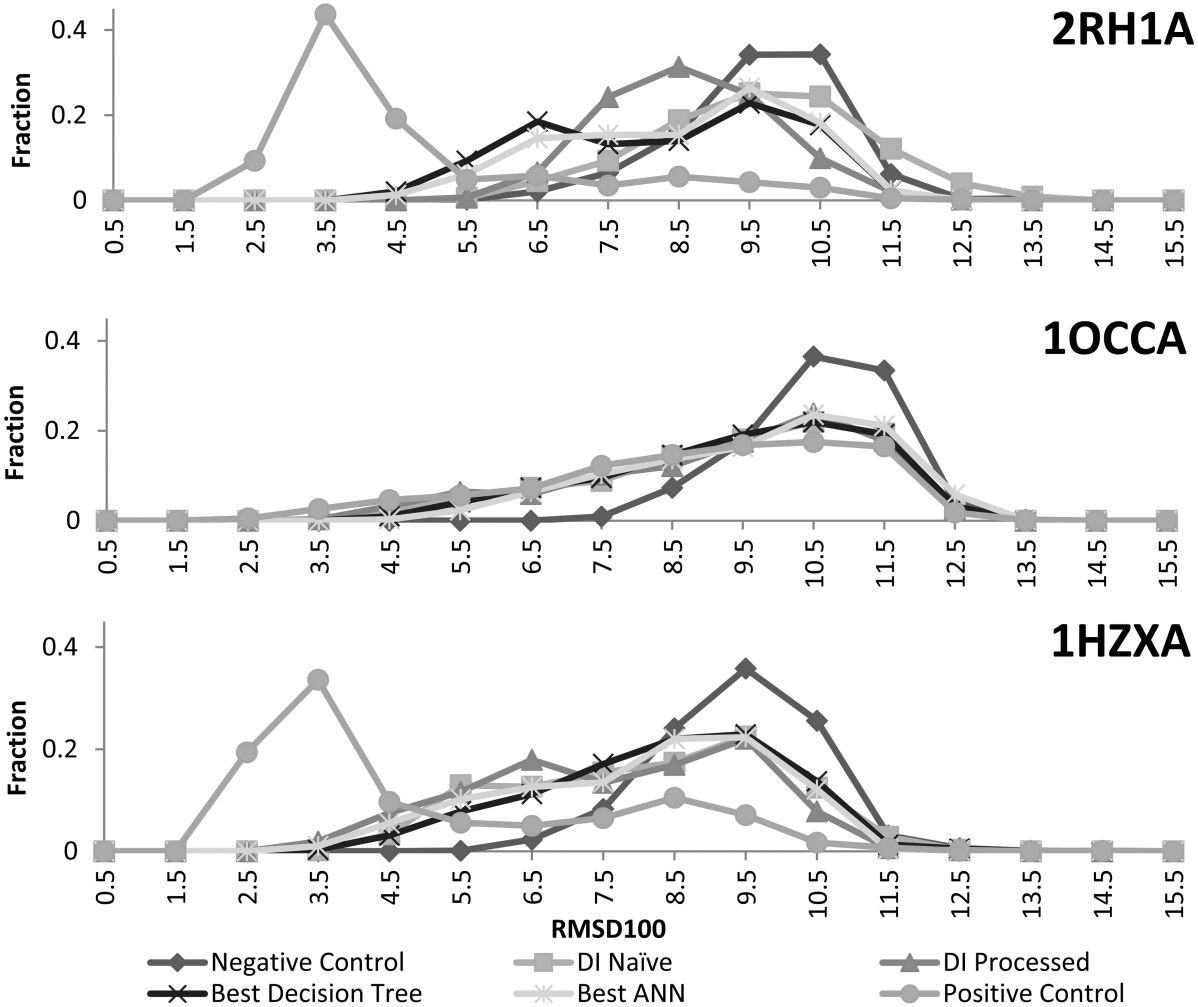
Comparison of protein model distribution across methods for 2RH1A, 1OCCA, and 1HZXA. The RMSD100 distributions of 1000 predicted models across naïve DI, processed DI, the best DTs, and the best ANNs above are book-ended by the distributions of the positive and negative controls. Contact restraints consistently shift the distributions towards lower RMSD100 models. There is little difference between methods for 1OCCA and 1HZXA. However, both machine learning methods shift more substantially towards lower RMSD100 values in the case of 2RH1A. One should also note that the distributions for all experimental methods approach that of the positive control for 1OCCA.

Having determined the upper limit of improvement, we compared the results of folding using the positive control restraint sets to the results obtained using the DI, DT, and ANN predicted contacts (Fig 5). All predictions were made using models with training sets that excluded data from the structures to be predicted. The usage of a set of diverse alpha-helical membrane proteins limits our possible set of training inputs to relatively few proteins as there are relatively few membrane protein families of known structure. We are further limited to proteins with alignments of sufficient depth to calculate DI for our coevolution-based contact prediction. While a larger training set would be desirable, we posit that the results we present are generalizable to other proteins.

The negative control shows the performance of BCL::Fold without contact restraints. All predicted contacts shift models towards lower RMSD, although not to the same degree as the improvement achieved with only correct contacts. However, the shift seen for cytochrome C oxidase (PDB: 1OCCA) is very close to the positive control. This is likely due to its especially high contact prediction accuracy. Both machine learning methods perform noticeably better than the DI based methods for beta2-adrenergic G protein-coupled receptor (PDB: 2RH1A). This is due to the methods’ automatic filtering of false positives between residues from opposite sides of the membrane. This improves accuracy from 12.4% (DI) to 30.7% (ANN) for the top L/2 contacts at a minimum separation of twelve.

**Fig 6:**
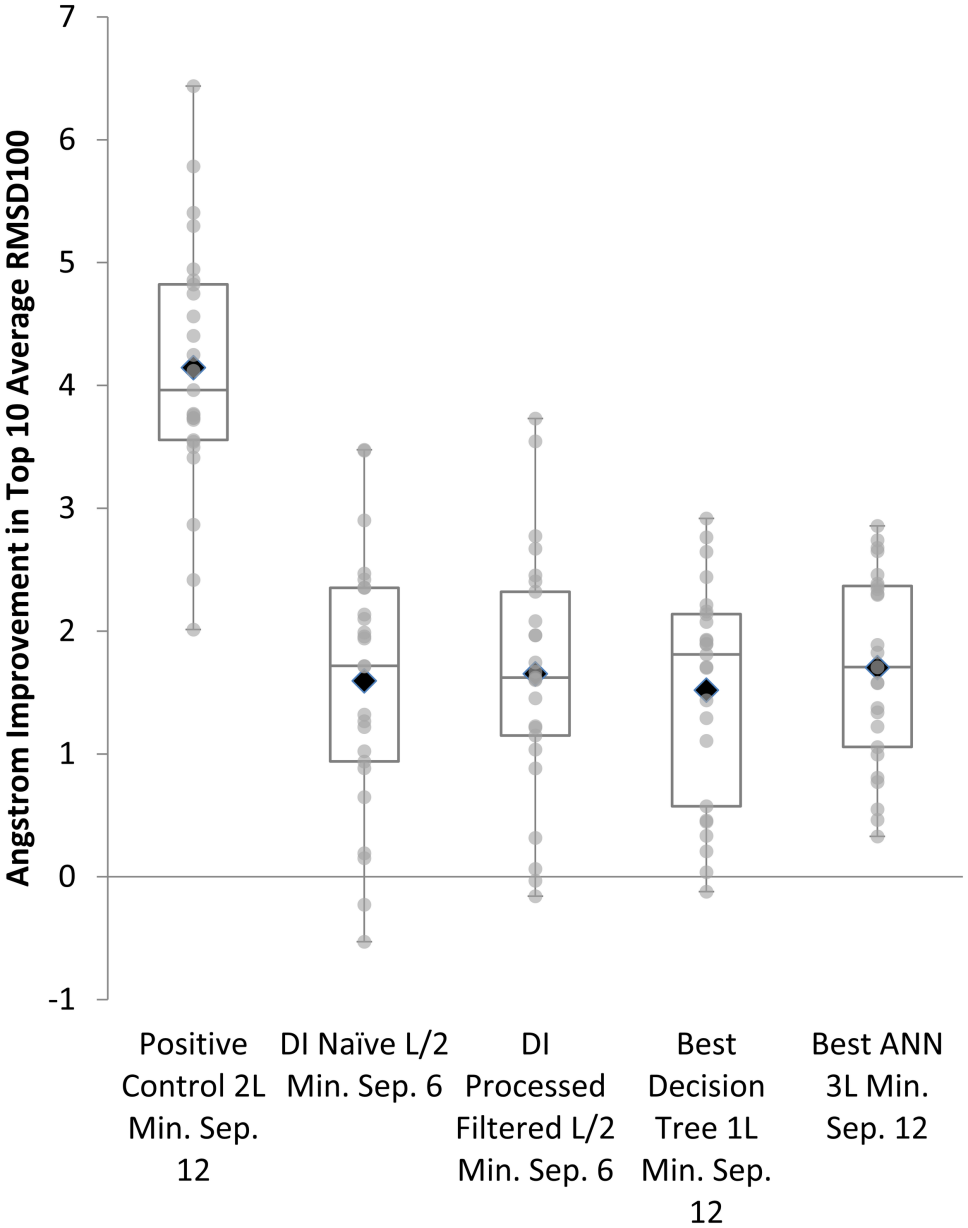
Influence of predicted contacts on folding accuracy. Box Plot Comparing Top 10 Models by Average Percent Improvement in RMSD100 across Benchmark Set for Best Direct Information, DT, and ANN Methods.

Correct contacts result in the greatest average improvement in RMSD100 by 4.1Å. Predicted contacts all provide improvement over the negative control, but are not statistically significantly different from one another as determined by the Wilcoxon signed-rank test (Fig 6). However, the distribution of improvements as gauged by the quartiles does appear to be consistently higher for ANNs. In addition, the average improvement is highest for results from structures predicted using the contacts from the best ANNs (1.7Å) compared to the second best, processed DI also at 1.7 Å. Tables 2 and 3 include full benchmark folding results across all categories. The mutual relationship of the folding results is invariant with respect to chosen structural similarity metric, for results using GDT-TS and TMscore, see S5 Table.

**Table 2:** Full benchmark folding results for controls and predicted restraints from the best methods—DI filtered, negative control, and positive control (Top 10 average RMSD100 Å).

PDBID	DI Filtered MSA Stats				Negative Best RMSD100			Pos. Control 2L ms 12		
	*L*	*TM*_*helix*_	*M*_*eff*_	*Cov*	Best	Top 10 Avg	S.D.	Best	Top 10 Avg	S.D.
**3NCYA**	**422**	12	1205	0.89	7.90	8.56	0.28	2.53	2.78	0.11
**2RH1A**	**442**	8	451	0.65	5.52	6.17	0.27	2.22	2.44	0.09
**1OKCA**	**292**	6	1043	0.89	6.45	7.61	0.42	3.56	3.89	0.17
**1XQFA**	**362**	11	1021	0.96	7.46	7.75	0.16	2.34	2.45	0.07
**3GD8A**	**223**	7	1062	0.96	6.25	6.58	0.20	2.88	3.08	0.13
**1L7VA**	**324**	10	1045	0.91	6.53	7.37	0.47	2.90	3.12	0.11
**3MKTA**	**460**	12	1068	0.92	6.33	7.93	0.57	3.86	4.17	0.20
**3RKON**	**473**	14	1722	0.83	7.80	8.30	0.29	2.86	3.36	0.21
**1OCCA**	**514**	12	754	0.98	7.33	7.71	0.23	2.32	2.85	0.32
**1OCCC**	**261**	6	521	0.96	7.44	8.00	0.21	5.36	5.59	0.08
**1PP9C**	**379**	8	581	0.92	7.15	7.59	0.22	3.56	4.04	0.27
**3H90A**	**283**	6	1039	0.97	6.62	7.26	0.37	3.17	3.84	0.24
**3B45A**	**180**	6	1073	0.87	6.30	6.53	0.12	3.46	3.66	0.10
**1PW4A**	**434**	12	1604	0.88	6.98	7.57	0.33	2.46	2.82	0.21
**3DHWA**	**203**	5	1065	0.88	6.94	7.52	0.30	5.33	5.51	0.11
**1YMGA**	**233**	7	1010	0.9	6.42	6.64	0.24	2.84	3.08	0.12
**3B60A**	**572**	6	1568	0.88	9.37	10.01	0.33	5.56	6.27	0.40
**2A65A**	**510**	12	1043	0.83	8.64	9.06	0.24	2.20	2.63	0.15
**1HZXA**	**340**	7	1151	0.8	5.77	6.28	0.23	2.08	2.16	0.06
**3PJZA**	**468**	12	923	0.79	7.69	8.59	0.36	4.14	4.82	0.35
**2XUTA**	**456**	14	1040	0.71	7.74	8.31	0.34	3.18	3.74	0.26
**3ZUXA**	**308**	10	1005	0.9	7.11	7.61	0.22	2.66	2.79	0.08
**2XQ2A**	**538**	15	1380	0.82	8.75	9.28	0.23	3.40	3.88	0.39
**3M71A**	**306**	10	646	0.97	5.88	6.41	0.34	2.34	2.45	0.08
**3QE7A**	**407**	14	1355	0.82	8.43	9.05	0.32	4.37	4.64	0.18
**Avg**	**376**	9.68	1055	0.875	7.15	7.75	0.29	3.26	3.60	0.18

The table displays the average, best, and standard deviation with respect to the RMSD100 across the entire benchmark set for positive and negative controls and with contacts predicted using filtered DI at L/2 and minimum separation of 6 (additional methods included in Table 3). In addition, each result row has the length (L), number of transmembrane helices (TMhelix), the effective alignment depth (Meff), and target sequence coverage (Cov) matched to each PDBID. The positive control shown was the best performing run analyzed and utilized 2L contacts at a minimum separation of 12.

**Table 3:** Full benchmark folding results for controls and predicted restraints from the best methods (Top 10 average RMSD100 Å).

PDBID	DI Naïve L/2 ms 6			DI Processed Filt L/2 ms 6			Best DT 1L ms 12			Best ANN 3L ms 12		
	Best	Top 10 Avg	S.D.	Best	Top 10 Avg	S.D.	Best	Top 10 Avg	S.D.	Best	Top 10 Avg	S.D.
**3NCYA**	**6.15**	**6.84**	0.39	6.17	6.89	0.47	6.26	6.85	0.38	6.66	7.19	0.26
**2RH1A**	5.84	5.98	0.08	5.49	5.86	0.23	4.26	**4.47**	0.10	**4.24**	4.60	0.21
**1OKCA**	5.12	5.90	0.32	5.49	5.99	0.30	5.28	5.47	0.13	**4.56**	**5.27**	0.26
**1XQFA**	5.87	6.81	0.51	**5.76**	6.53	0.38	5.81	**6.46**	0.31	6.01	6.70	0.27
**3GD8A**	3.58	**3.68**	0.06	4.08	4.26	0.11	3.62	3.82	0.13	**3.54**	3.72	0.09
**1L7VA**	**5.19**	6.34	0.54	5.71	**6.33**	0.33	6.09	6.91	0.41	6.31	6.82	0.24
**3MKTA**	6.27	6.67	0.14	5.65	5.85	0.12	6.11	6.50	0.24	**4.73**	**5.57**	0.39
**3RKON**	6.11	6.98	0.48	5.69	6.34	0.31	7.17	7.86	0.30	**4.98**	**5.85**	0.38
**1OCCA**	3.94	4.23	0.22	**3.84**	**4.16**	0.20	4.45	4.79	0.17	4.46	5.06	0.27
**1OCCC**	5.63	**6.06**	0.22	6.20	6.39	0.16	6.02	6.19	0.13	**5.52**	6.18	0.24
**1PP9C**	5.26	5.63	0.21	5.30	5.62	0.20	**5.04**	**5.51**	0.23	6.41	6.81	0.22
**3H90A**	**4.78**	**4.90**	0.09	5.71	5.80	0.05	5.05	5.33	0.14	4.79	4.95	0.09
**3B45A**	**4.29**	**4.54**	0.12	4.64	4.93	0.17	4.43	4.63	0.15	4.70	4.82	0.10
**1PW4A**	5.20	5.47	0.17	**4.50**	**5.11**	0.34	5.35	5.67	0.23	5.78	6.23	0.23
**3DHWA**	6.41	6.63	0.11	**5.81**	6.30	0.21	6.88	7.19	0.13	5.91	**6.30**	0.21
**1YMGA**	**3.89**	**4.22**	0.18	4.06	4.23	0.12	4.29	4.43	0.06	3.99	4.25	0.16
**3B60A**	8.69	9.36	0.32	**8.57**	**8.86**	0.18	9.84	10.13	0.23	9.25	9.55	0.11
**2A65A**	4.78	5.59	0.37	**4.67**	**5.33**	0.32	6.02	6.42	0.29	5.72	6.32	0.36
**1HZXA**	3.95	4.14	0.16	3.31	3.61	0.17	3.64	4.12	0.20	**3.28**	**3.60**	0.16
**3PJZA**	8.01	8.44	0.18	7.95	8.53	0.25	7.41	8.02	0.32	**6.42**	**7.02**	0.33
**2XUTA**	8.34	8.84	0.28	8.19	8.47	0.16	8.00	8.27	0.12	**6.73**	**7.50**	0.42
**3ZUXA**	4.88	5.14	0.14	4.50	**4.84**	0.21	4.87	5.17	0.21	**4.33**	5.31	0.53
**2XQ2A**	**8.27**	9.51	0.48	9.12	9.32	0.10	8.44	9.08	0.35	8.38	**8.96**	0.27
**3M71A**	4.69	**5.19**	0.28	**4.56**	5.53	0.35	4.88	5.31	0.18	4.57	5.42	0.37
**3QE7A**	**5.98**	**6.69**	0.40	6.23	7.30	0.43	6.81	7.12	0.21	6.13	7.16	0.56
**Avg**	5.64	6.15	0.26	5.65	6.09	0.24	5.84	6.23	0.21	**5.50**	**6.05**	0.27

The table displays the average, best, and standard deviation with respect to the RMSD100 across the entire benchmark set for contacts predicted from one of the following methods: naïve DI at L/2 and minimum separation of 6, processed, best DT at 1L and minimum separation of 12, or best ANN at 3L and a minimum separation of 12. In addition, each result row has the length (L), number of transmembrane helices (TMhelix), the effective alignment depth (Meff), and target sequence coverage (Cov) matched to each PDBID. The L-fraction and minimum separation for each method was independently optimized by sampling across L-fractions of L/10, L/5, L/2, 1L, 2L, and 3L as well as minimum separations of 6 or 12. Further alignment details are in Table 1. Additionally, the best single model and top 10 average results from all included contact prediction methods for each PDBID is bolded. Our method using ANNs has both the lowest average RMSD100 for the best model (5.50Å) as well as the lowest top 10 average across the benchmark (6.05Å).

**Fig 7:**
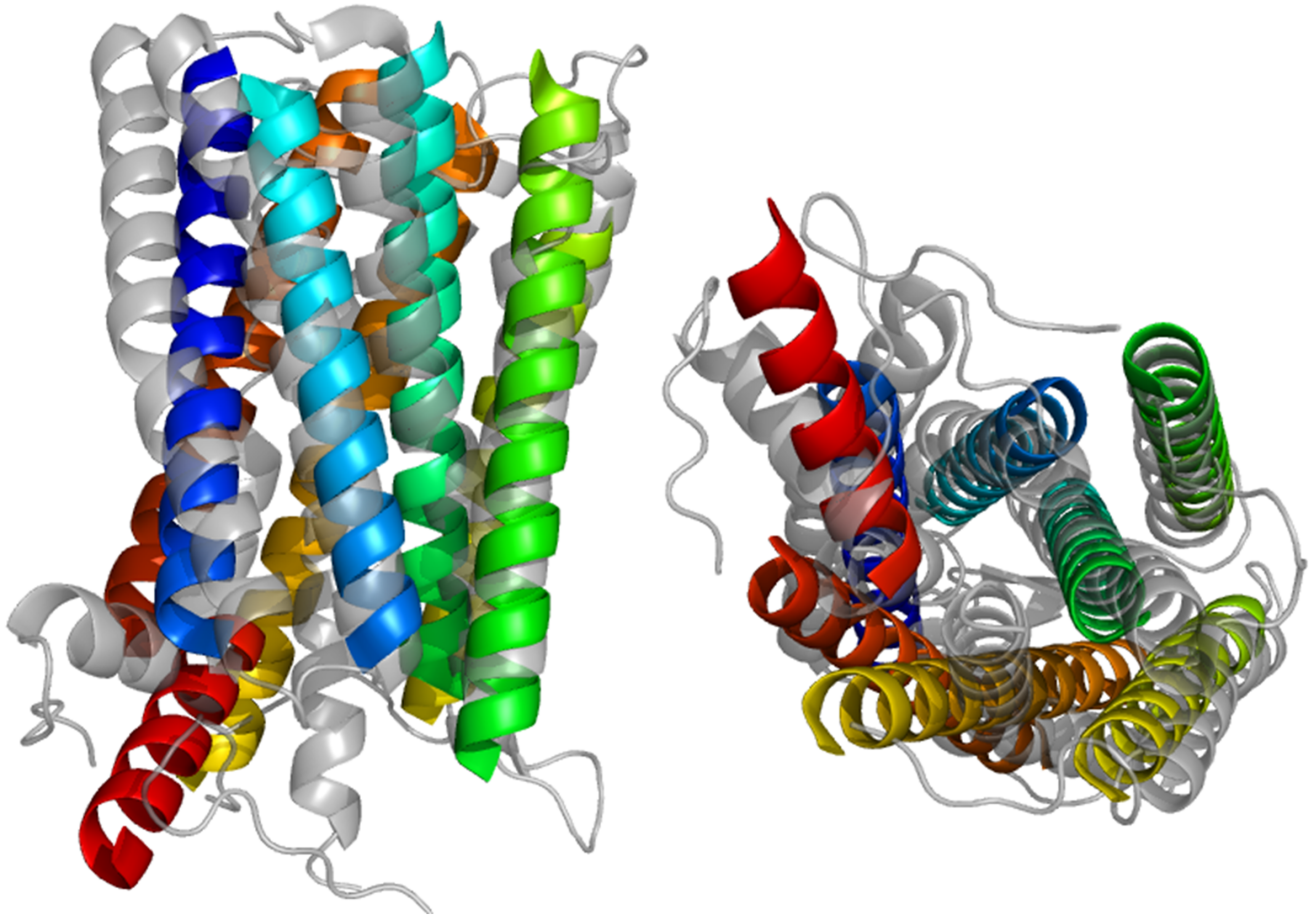
Contact assisted prediction of 1HZXA structure. Visualization of Best Protein Model by RMSD100 Aligned to Native from Contact Predictions Made by the Best ANN (3L and Minimum Separation 12).

Fig 7 shows how close the best model by RMSD100 replicates the native fold for 1HZXA (3.3Å). The topology is correct and there is substantial superimposition of model helices with those of the native structure. One expects some deviation as BCL::Fold uses idealized helices with limited bends or kinks. These models still require the addition of side chains as well as other refinement but the similarity between the predicted model and the native greatly simplifies refinement and final all-atom prediction.

The best model by RMSD100 (3.3Å) is aligned above to the native structure. The model was produced as part of a folding run of 1000 proteins using the top 3L contacts as predicted by the best ANN with a minimum sequence separation of 12. Helices line up well, as can be seen from both an in-membrane and above-membrane view. Deviation from the native structure is due in part to the use of idealized SSEs that do not bend.

## Conclusion

Membrane protein *de-novo* structure prediction can be aided by predicted contacts, which is of particular importance given the challenges of experimental MP structure determination. The evolutionary methods for contact prediction, which use rapidly growing pool of protein sequence information can be expected to render more proteins (including MPs) amenable to analysis and hence improve our understanding of MP structure. Substantial improvement is possible by increasing the accuracy of contact prediction methods as evidenced by the correct contact results. One may attain further improvement by leveraging the confidence in each contact prediction or dynamically adjusting the contact restraints used based on scoring and confidence values. Since development of this method, new, improved methods for contact prediction have emerged [20, 36, 43], which allow to further increase the applicability of approaches similar to the one we described. Despite use of imperfectly predicted contacts, we observed a significant improvement in contact-restrained model accuracy, in comparison to the unrestrained ones.

Co-evolutionary methods for coupling inference suffer from systematic biases, which can be alleviated by machine learning. While this class of methods appears to work well for contact prediction, their goal only partially overlaps with the goal of contact prediction. Not all contacting residues co-evolve and—conversely—not all co-evolving residues contact. For membrane proteins, the most notable class of inferred couplings that do not correspond to contacts is the couplings between termini of transmembrane spans. Use of prior information, either on the stage of inference, or post-processing with machine learning does alleviate some of these “mispredictions”, thus improving the expected utility of these methods for protein structure prediction.

The accuracy of protein folding by BCL::Fold improves significantly by use of experimental and predicted contacts. As simulating protein folding by macromolecular simulations (molecular dynamics) is still largely unfeasible, the most promising results are to be achieved by stochastic methods that allow for sampling larger chunks of conformational space [34, 44]. The assembly of large structural elements, an approach implemented in BCL::Fold, permits for reaching native-like topologies of predicted models with a fewer computational steps. Use of sparse experimental information [42, 45] or prior information on protein structure—be it experimental or predicted—does further increase the likelihood of sampling and retaining the models with correct (close to the native) folds.

BCL::Fold’s protein structure prediction performance is best with L-fractions of L/2 and higher (S1 Fig). Machine learning methods achieve the best average benchmark accuracies for L-fractions most suitable for protein fold prediction using BCL::Fold. Thus, machine learning methods improve protein fold prediction.

We strongly believe that with improvement in evolutionary contact prediction methodology, increase of sequence information available and introduction of model refinement techniques to BCL::Fold (e.g. aggressive secondary structure bending, optimization of hydrogen bonding etc.), the approach described above will become a feasible strategy for accurate membrane protein structure prediction.

## Availability

BCL::Fold is implemented as part of the BioChemical Library, a suite of software currently under development in the Meiler laboratory (http://www.meilerlab.org/index.php/bclcommons/show/b_apps_id/1). BCL software, including BCL::Fold, is freely available for academic use. Predicted contacts and top 10 structures are available at DOI 10.6084/m9.figshare.4600372. All source PDB files are available from the RCSB database http://www.rcsb.org/ and homologous sequences via BLAST https://blast.ncbi.nlm.nih.gov/Blast.cgi.

## Supporting information

S1 Fig RMSD100 distribution of predicted models as increasing L-fractions of known contacts are used, related to experimental procedures. This set of proteins represents the range of improvement in model distributions from most to least drastic (2RH1A, 3GD8A, and 1OCCA). We depicted distributions for 2RH1A, 3GD8A, and 1OCCA in darker colors as the number of contacts increases. 2RH1A has a drastic shift from a peak at 9.5Å to 3.5Å. 3GD8A represents an intermediate improvement where the distribution becomes bimodal, with the original peak diminished at 10.5Å but not to the extent of 2RH1A. There is also a new peak at 3.5Å. Finally, 1OCCA has a peak which does not shift substantially but the tail is shifted towards lower RMSD100 scores, indicating some improved sampling with increasing numbers of contact restraints.
(DOCX)

S2 Fig L-fraction optimization for structure prediction using contacts from the positive control, naïve direct information, best decision tree, and best ANN, related to Table 3. This is the average RMSD100 improvement for the top 10 models across the L-fractions examined with a minimum separation of 6 and 12 (light and dark colors respectively) for the positive control (black), naïve DI (red), best decision tree (green), and the best ANN (blue). This optimization was done with a random subset of 9 of the 25 benchmark proteins due to computational limitations. Using known contacts leads to greater improvement, which plateaus at 2L and a maximum average improvement with a minimum separation of 12 of 2.90Å. Naïve DI, and the best decision tree peak for L-fractions of L/2 and 1L at a minimum separation of 6 and 12 respectively (1.81Å for both). Finally, the best ANN peaks for the maximum L-fraction of 3L and a minimum separation of 12 with a maximum average improvement of 2.05Å.
(DOCX)

S3 Fig AUC and integral for precision across fraction predicted positive after all 29 rounds of ANN weights-based optimization, related to Fig 1. In addition to the input sensitivity iteration method used with decision trees, we also attempted a descriptor optimization that determines which descriptors are most useful by analyzing the weights of the ANN models trained. We graphed the results of a 29 round optimization for both the AUC and the integral of the positive predictive value across the fraction predicted positive from 0.01% to 0.55%. As we remove descriptors, the AUC slowly trends upwards. There is a slight plateau once one reaches 203 descriptors. However, the positive predictive value integral is largely steady within a range until one reaches 146 descriptors. This is the highest point, followed by several higher but declining values as one approaches the final round of optimization. Given that the positive predictive value integral is more representative of the top L contact predictions desired for protein fold prediction, we used the top 146 descriptors for final contact prediction (round 23).
(DOCX)

S4 Fig High, medium, and low accuracy top L/10 visualized contacts for 3GD8A, 3MKTA, and 2RH1A respectively (DI-only filtered), related to Table 2. This is an example of the top L/10 predicted contacts for 3GD8A (left) showing both the distribution across the protein structure and the potential accuracy of the top DI pairs for contact prediction. The accuracy is approximately 91%. For this set, only two pairs are incorrect at 9.2Å and 18.1Å. The latter is a predicted contact between flexible loop regions. 3MKTA (middle) shows the distribution across the protein structure and is an example of the medium accuracy possible for the top DI pairs. Accuracy for this protein is 56.5%. Few pairs are incorrect, and most of them are within the 8-11Å range. Many of the incorrect pairs are still “near-contacts”. 2RH1A (right) shows one of the few examples of low accuracy in the absence of topology prediction assistance. Blue lines connect correct contact pairs (within the 8Å threshold). Yellow lines connect incorrect pairs that are “near-contacts” (between 8Å and 12Å). Red lines connect incorrect pairs. Accuracy for top L/10 is approximately 18.9%.
(DOCX)

S5 Fig Topology-based filtering threshold selection, related to Fig 2. To determine the optimal difference threshold for filtering contacts to maximize accuracy we evaluated thresholds from zero to 0.95 for all top L-fractions and minimum separations of 6 and 12. Accuracy decreases initially for overly stringent cutoffs (near zero), which eliminate too many possible contacts for most sets of parameters. Values begin improving for most L-fractions and minimum separation values beginning around a maximum predicted transmembrane separation of 0.2. This improvement is optimal around 0.35 across L-fractions and as such, the filtering threshold was set to a maximum predicted vertical separation of 0.35. As the threshold increases beyond 0.35, the beneficial effects of topology prediction decrease slowly towards zero. The top 1L contact predictions decreases beyond zero during this latter range likely due to a clustering of contacts with inaccurate transmembrane separation values of 1.0 due to incorrect topology predictions in the range between the top L/2 and L contacts.
(DOCX)

S6 Fig RMSD-RMSD comparison of the top 10 models from runs with contact restraints from the best model (ANN) and without any contact restraints, related to Fig 6. The plot shows the relative benefit of using predictions from the best model, the optimized ANN at 3L minimum separation of 12, in comparison to folding without any contact restraints. Average RMSD100 for the top 10 models in each run are plotted such that the result from the negative control set is given on the x-axis and the set using the best predictions is on the y-axis. Equal performance is depicted via the dotted x = y line and any point below this diagonal is improved by inclusion of our predicted restraints. All 25 points are below the diagonal showing consistent improvement across the benchmark set.
(DOCX)

S7 Fig Comparison of direct information, running accuracy, and confidence weights across the top 1L contacts for 3MKTA and 1OCCA, related to experimental procedures. We have included a comparison of the direct information values, running accuracy, and confidence weighting for 3MKTA, an average example of good contact prediction using direct information, and 1OCCA, a poorly performing example from an early direct information based method. The distribution and magnitude of the confidence weighting is relatively similar, while the direct information values are much larger and decrease more slowly for 3MKTA. The point where confidence weighting crosses the 1.0 threshold—distinguishes between the set of contacts weighted more and less heavily by confidence scoring. The accuracy for 3MKTA is much higher initially and stays around 30% across the entire set of the top L contact predictions. 1OCCA’s accuracy drops off much more precipitously and quickly approaches 0%. Thus, the confidence based score increases the weighting for many contacts that are in a range at or below 10% accuracy.
(DOCX)

S1 File Supplementary information. This file includes supplementary information and explanations.
(PDF)

S1 Table Categories of global, sequence, and direct information (Correlation) descriptors, related to experimental procedures. The table above contains all the categories of descriptors initially analyzed. They are divided into the three broad categories. The first is global position descriptors—the location of each element of the pair i,j being predicted within the context of the sequence. The second category is the sequence descriptors—biochemical, BLAST, and predicted secondary structure information regarding the amino acids as well as aggregated descriptors (mean and standard deviation) calculated across the entire sequence for the given properties and BLAST data. Finally, the third lists coupling descriptors, which includes various aggregated descriptors such as the max, mean, sequence normalized mean, standard deviation, and sum across collections of the elemental correlation descriptors (by window or across e-value/filtering parameters).
(DOCX)

S2 Table Top 30 descriptors used for best DT model, related to Fig 1. This is the final set of 30 descriptors selected for use with DTs to predict long-range contacts. We determined this set using an iterative process whereby we scored the usefulness of descriptors for a given model type initially using F-score and then input sensitivity. We evaluated models using increasing subsets of the ranked descriptors. We then selected optimal thresholds and repeated the scoring and evaluation process until improvement plateaued or decreased. The top 30 descriptors are described above along with the type of descriptor and the input sensitivity at the last iteration.
(DOCX)

S3 Table Table of aggregated contact prediction accuracies from best methods across categories and optimal L-fractions for each included method. This table lists the final set of accuracies for the best model from each method category (naïve DI, processed filtered DI, DTs, ANNs) with all optimal L-fractions for the given methods as well as L/10 at a minimum separation of 12 to show some of the highest precision prediction sets. All include the length (L), number of transmembrane helices (TM_helix_), the effective alignment depth (M_eff_), and target sequence coverage (Cov) matched to each PDBID for comparison. Additionally, the best accuracy for each PDBID and L-fraction is bolded. DTs outperform all other methods at L/10 and a minimum separation of 12 (45.0%) and at L/2 and a minimum separation of 6 (29.4%). ANNs outperform all other methods at 1L and a minimum separation 12 (24.0%) and at 3L and a minimum separation of 12 (15.3%).
(DOCX)

S4 Table Mean positive predictive value of underlying method (DCA), proposed methods (DT: decision trees, ANN: artificial neural networks) and three state-of-art methods (PSICOV, CCMpred and FreeContact). Comparison across methods using positive predictive value of underlying method (DCA), proposed methods (DT: decision trees, ANN: artificial neural networks) and three state-of-art methods (PSICOV, CCMpred and FreeContact) at three separation cut-offs (6+, 12+ and 24+ residues), as well as 4 inclusion thresholds (L/10, L/5, L/2, L, where L is the length of protein). All results are based on the same alignments. We would like to emphasize, that this method is based on a first-generation mean field DCA implementation. Current methods for evolutionary coupling analysis take advantage of known improvements in the field in form of better sequence reweighting, use of regularization instead of pseudocounts and inferring more appropriate statistical models (Potts models instead of Ising models of original DCA). To facilitate comparison, we have based all the predictions (both by our method and the others) on the same alignments. We posit, that the methods we propose should be successfully applicable to the coupling inference methods of newer generations.
(DOCX)

S5 Table Folding results using alternate similarity metrics GDT-TS and TMscore. Here we have replicated Table 3 of main manuscript containing the same results, but with GDT-TS and TMscore as similarity metrics. While not as wide-spread as TM-score or GDT-TS, RMSD100 is also a metric for length-independent comparison of structural similarity and enables comparison to prior work using BCL::Fold.
(DOCX)

## References

[1] Dill KA, MacCallum JL, The protein-folding problem, 50 years on. Science, 2012 338(6110): p. 1042–6. 10.1126/science.1219021 23180855

[2] Raman P, Cherezov V, Caffrey M, The Membrane Protein Data Bank. Cell Mol Life Sci, 2006 63(1): p. 36–51. 10.1007/s00018-005-5350-6 16314922 PMC2792347

[3] Fox NK., Brenner SE, Chandonia JM, SCOPe: Structural Classification of Proteins—extended, integrating SCOP and ASTRAL data and classification of new structures. Nucleic Acids Res, 2014 42(Database issue): p. D304–9. 10.1093/nar/gkt1240 24304899 PMC3965108

[4] Berman HM, Westbrook J, Feng Z, Gilliland G, Bhat TN, Weissig H et al, The Protein Data Bank. Acta Crystallogr D Biol Crystallogr, 2002 58(Pt 6 No 1): p. 899–907. 12037327 10.1107/s0907444902003451

[5] Tusnády GE, Dosztányi Z, Simon I, PDB_TM: selection and membrane localization of transmembrane proteins in the protein data bank. Nucleic Acids Res, 2005 33(Database issue): p. D275–8. 10.1093/nar/gki002 15608195 PMC539956

[6] Ahram M, Litou ZI, Fang R, Al-Tawallbeh G, Estimation of membrane proteins in the human proteome. In Silico Biol, 2006 6(5): p. 379–86. 17274767

[7] Bakheet TM, Doig AJ, Properties and identification of human protein drug targets. Bioinformatics, 2009 25(4): p. 451–7. 10.1093/bioinformatics/btp002 19164304

[8] Graña O, Baker D, MacCallum RM, Meiler J, Punta M, Rost B et al, CASP6 assessment of contact prediction. Proteins, 2005 61 Suppl 7: p. 214–24. 16187364 10.1002/prot.20739

[9] Ezkurdia I, Grana O, Izarzugaza JM, Tress ML, Assessment of domain boundary predictions and the prediction of intramolecular contacts in CASP8. Proteins, 2009 77 Suppl 9: p. 196–209. 19714769 10.1002/prot.22554

[10] Izarzugaza JM, Grana O, Tress ML, Valencia A, Clarke ND, Assessment of intramolecular contact predictions for CASP7. Proteins, 2007 69 Suppl 8: p. 152–8. 17671976 10.1002/prot.21637

[11] Monastyrskyy B, D'Andrea D, Fidelis K, Tramontano A, Kryshtafovych A, Evaluation of residue-residue contact prediction in CASP10. Proteins, 2013. 10.1002/prot.24340 PMC3823628 23760879

[12] Di Lena P, Nagata K, Baldi P, Deep architectures for protein contact map prediction. Bioinformatics, 2012 28(19): p. 2449–2457. 10.1093/bioinformatics/bts475 22847931 PMC3463120

[13] Fuchs A, Kirschner A, Frishman D, Prediction of helix-helix contacts and interacting helices in polytopic membrane proteins using neural networks. Proteins, 2009 74(4): p. 857–71. 10.1002/prot.22194 18704938

[14] Zhang H, Huang Q, Bei Z, Wei Y, Floudas CA, COMSAT: Residue contact prediction of transmembrane proteins based on support vector machines and mixed integer linear programming. Proteins, 2016 84(3): p. 332–48. 10.1002/prot.24979 26756402

[15] Karakaş M, Woetzel N, Meiler J, BCL::contact-low confidence fold recognition hits boost protein contact prediction and de novo structure determination. J Comput Biol, 2010 17(2): p. 153–68. 10.1089/cmb.2009.0030 19772383 PMC3148831

[16] Weigt M, White RA, Szurmant H, Hoch JA, Hwa T, Identification of direct residue contacts in protein-protein interaction by message passing. Proc Natl Acad Sci U S A, 2009 106(1): p. 67–72. 10.1073/pnas.0805923106 19116270 PMC2629192

[17] Morcos F, Pagnani A, Lunt B, Bertolino A, Marks DS, Sander C et al Direct-coupling analysis of residue coevolution captures native contacts across many protein families. Proc Natl Acad Sci U S A, 2011 108(49): p. E1293–301. 10.1073/pnas.1111471108 22106262 PMC3241805

[18] Feinauer C, Skwark MJ, Pagnani A, Aurell E, Improving contact prediction along three dimensions. PLoS Comput Biol, 2014 10(10): p. e1003847 10.1371/journal.pcbi.1003847 25299132 PMC4191875

[19] Aurell E, The Maximum Entropy Fallacy Redux? PLoS Comput Biol, 2016 12(5): p. e1004777 10.1371/journal.pcbi.1004777 27171259 PMC4865147

[20] Jones DT, Singh T, Kosciolek T, Tetchner S, MetaPSICOV: combining coevolution methods for accurate prediction of contacts and long range hydrogen bonding in proteins. Bioinformatics, 2015 31(7): p. 999–1006. 10.1093/bioinformatics/btu791 25431331 PMC4382908

[21] Finn RD, Mistry J, Tate J, Coggill P, Heger A, Pollington JE et al, The Pfam protein families database: towards a more sustainable future. Nucleic acids research, 2016 44(D1): p. D279–D285. 10.1093/nar/gkv1344 26673716 PMC4702930

[22] Hopf TA, Colwell LJ, Sheridan R, Rost B, Sander C, Marks DS, Three-dimensional structures of membrane proteins from genomic sequencing. Cell, 2012 149(7): p. 1607–21. 10.1016/j.cell.2012.04.012 22579045 PMC3641781

[23] Skwark MJ, Abdel-Rehim A, Elofsson A, PconsC: combination of direct information methods and alignments improves contact prediction. Bioinformatics, 2013 29(14): p. 1815–6. 10.1093/bioinformatics/btt259 23658418

[24] Skwark MJ, Raimondi D, Michel M, Elofsson A, Improved contact predictions using the recognition of protein like contact patterns. PLoS Comput Biol, 2014 10(11): p. e1003889 10.1371/journal.pcbi.1003889 25375897 PMC4222596

[25] Mistry J, Finn RD, Eddy SR, Bateman A, Punta M, Challenges in homology search: HMMER3 and convergent evolution of coiled-coil regions. Nucleic Acids Res, 2013 41(12): p. e121 10.1093/nar/gkt263 23598997 PMC3695513

[26] Marks DS, Colwell LJ, Sheridan R, Hopf TA, Pagnani A, Zecchina R et al, Protein 3D Structure Computed from Evolutionary Sequence Variation. PLoS One, 2011 6(12): p. e28766 10.1371/journal.pone.0028766 22163331 PMC3233603

[27] Remmert M, Biegert A, Hauser A, Söding J, HHblits: lightning-fast iterative protein sequence searching by HMM-HMM alignment. Nat Methods, 2012 9(2): p. 173–5. 10.1038/nmeth.1818 22198341

[28] Viklund H, Bernsel A, Skwark M, Elofsson A, SPOCTOPUS: a combined predictor of signal peptides and membrane protein topology. Bioinformatics, 2008 24(24): p. 2928–9. 10.1093/bioinformatics/btn550 18945683

[29] Viklund H and Elofsson A, OCTOPUS: improving topology prediction by two-track ANN-based preference scores and an extended topological grammar. Bioinformatics, 2008 24(15): p. 1662–8. 10.1093/bioinformatics/btn221 18474507

[30] Koehler-Leman J, Mueller R, Karakas M, Woetzel N, Meiler J, Simultaneous prediction of protein secondary structure and transmembrane spans. Proteins, 2013 81(7): p. 1127–40. 10.1002/prot.24258 23349002 PMC5064873

[31] Zurada JM, Malinowski A, Usui S, Perturbation method for deleting redundant inputs of perceptron networks. Neurocomputing, 1997 14(2): p. 177–193.

[32] Engelbrecht AP, Sensitivity analysis for selective learning by feedforward neural networks. Fundamenta Informaticae, 2001 46(3): p. 219–252.

[33] Woetzel N, Karakaş M, Staritzbichler R, Müller R, Weiner BE, Meiler J, BCL::Score—knowledge based energy potentials for ranking protein models represented by idealized secondary structure elements. PLoS One, 2012 7(11): p. e49242 10.1371/journal.pone.0049242 23173051 PMC3500277

[34] Karakaş M, Woetzel N, Staritzbichler R, Alexander N, Weiner BE, Meiler J, BCL::Fold—de novo prediction of complex and large protein topologies by assembly of secondary structure elements. PLoS One, 2012 7(11): p. e49240 10.1371/journal.pone.0049240 23173050 PMC3500284

[35] Weiner BE, Woetzel N, Karakaş M, Alexander N, Meiler J, BCL::MP-fold: folding membrane proteins through assembly of transmembrane helices. Structure, 2013 21(7): p. 1107–17. 10.1016/j.str.2013.04.022 23727232 PMC3738745

[36] Baldassi C, Zamparo M, Feinauer C, Procaccini A, Zecchina R, Weigt et al, Fast and Accurate Multivariate Gaussian Modeling of Protein Families: Predicting Residue Contacts and Protein-Interaction Partners. Plos One, 2014 9(3). 10.1371/journal.pone.0092721 PMC3963956 24663061

[37] Kent JT, Information gain and a general measure of correlation. Biometrika, 1983 70(1): p. 163–173.

[38] Dekker JP, Fodor A, Aldrich RW, Yellen G A perturbation-based method for calculating explicit likelihood of evolutionary co-variance in multiple sequence alignments. Bioinformatics, 2004 20(10): p. 1565–1572. 10.1093/bioinformatics/bth128 14962924

[39] Jones DT, Buchan DW, Cozzetto D, Pontil M, PSICOV: precise structural contact prediction using sparse inverse covariance estimation on large multiple sequence alignments. Bioinformatics, 2012 28(2): p. 184–90. 10.1093/bioinformatics/btr638 22101153

[40] Seemayer S, Gruber M, Söding J., CCMpred—fast and precise prediction of protein residue-residue contacts from correlated mutations. Bioinformatics, 2014 30(21): p. 3128–30. 10.1093/bioinformatics/btu500 25064567 PMC4201158

[41] Kaján L, Hopf TA, Kalaš M, Marks DS, Rost B, FreeContact: fast and free software for protein contact prediction from residue co-evolution. BMC Bioinformatics, 2014 15: p. 85 10.1186/1471-2105-15-85 24669753 PMC3987048

[42] Weiner BE, Alexander N, Akin LR, Woetzel N, Karakas M, Meiler J, BCL::Fold—protein topology determination from limited NMR restraints. Proteins, 2014 82(4): p. 587–95. 10.1002/prot.24427 24123100 PMC3949166

[43] Feinauer C, Skwark MJ, Pagnani A, Aurell E, Improving Contact Prediction along Three Dimensions. Plos Computational Biology, 2014 10(10). 10.1371/journal.pcbi.1003847 PMC4191875 25299132

[44] Leaver-Fay A, Tyka M, Lewis SM, Lange OF, Thompson J, Jacak R et al, ROSETTA3: an object-oriented software suite for the simulation and design of macromolecules. Methods Enzymol, 2011 487: p. 545–74. 10.1016/B978-0-12-381270-4.00019-6 21187238 PMC4083816

[45] Fischer AW, Alexander NS, Woetzel N, Karakas M, Weiner BE, Meiler J, BCL::MP-Fold: Membrane protein structure prediction guided by EPR restraints. Proteins, 2015 83(11): p. 1947–62. 10.1002/prot.24801 25820805 PMC5064833

