# Supplementary material for: Membrane protein contact and structure prediction using co-evolution in conjunction with machine learning
Source: PLoS One. 2017 May 24;12(5):e0177866. doi: 10.1371/journal.pone.0177866 (PMC5443516; doi:10.1371/journal.pone.0177866)
Supplement: S1 Fig — This set of proteins represents the range of improvement in model distributions from most to least drastic (2RH1A, 3GD8A, and 1OCCA). We depicted distributions for 2RH1A, 3GD8A, and 1OCCA in darker colors as the number of contacts increases. 2RH1A has a drastic shift from a peak at 9.5Å to 3.5Å. 3GD8A represents an intermediate improvement where the distribution becomes bimodal, with the original peak diminished at 10.5Å but not to the extent of 2RH1A. There is also a new peak at 3.5Å. Finally, 1OCCA has a peak which does not shift substantially but the tail is shifted towards lower RMSD100 scores, indicating some improved sampling with increasing numbers of contact restraints. (DOCX) [file pone.0177866.s001.docx]

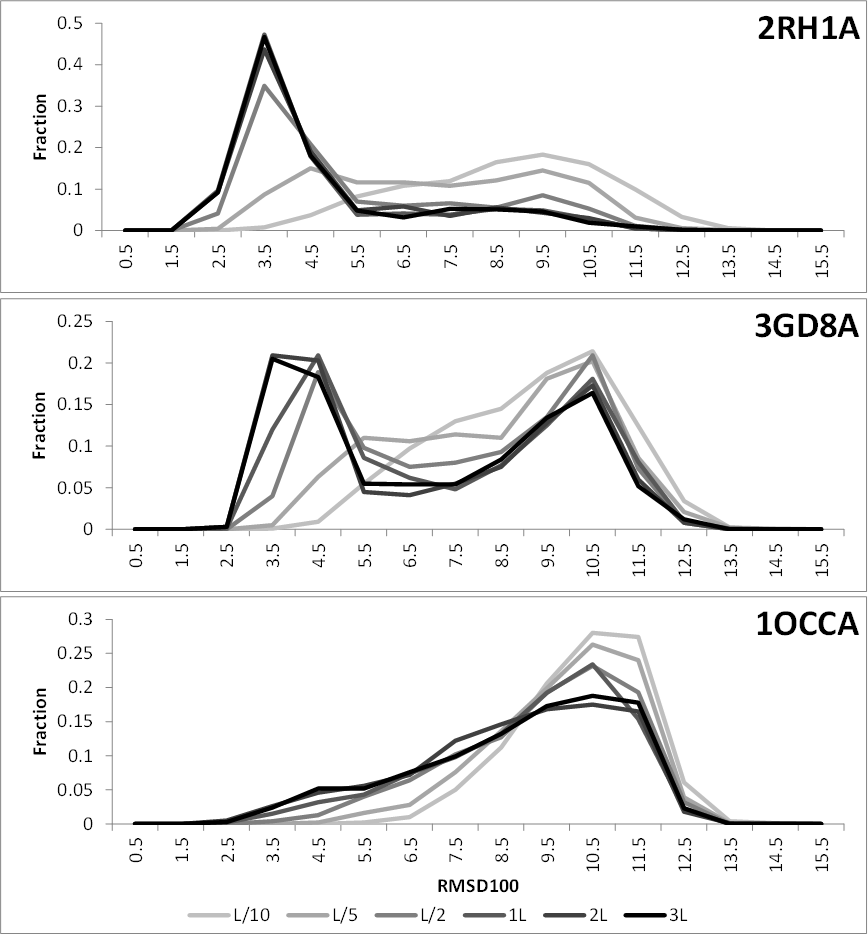


S1 Fig. RMSD100 Distribution of Predicted Models as Increasing L-Fractions of Known Contacts are Used, Related to Experimental Procedures.

This set of proteins represents the range of improvement in model distributions from most to least drastic (2RH1A, 3GD8A, and 1OCCA). We depicted distributions for 2RH1A, 3GD8A, and 1OCCA in darker colors as the number of contacts increases. 2RH1A has a drastic shift from a peak at 9.5Å to 3.5Å. 3GD8A represents an intermediate improvement where the distribution becomes bimodal, with the original peak diminished at 10.5Å but not to the extent of 2RH1A. There is also a new peak at 3.5Å. Finally, 1OCCA has a peak which does not shift substantially but the tail is shifted towards lower RMSD100 scores, indicating some improved sampling with increasing numbers of contact restraints.
