# Supplementary material for: Membrane protein contact and structure prediction using co-evolution in conjunction with machine learning
Source: PLoS One. 2017 May 24;12(5):e0177866. doi: 10.1371/journal.pone.0177866 (PMC5443516; doi:10.1371/journal.pone.0177866)
Supplement: S2 Fig — This is the average RMSD100 improvement for the top 10 models across the L-fractions examined with a minimum separation of 6 and 12 (light and dark colors respectively) for the positive control (black), naïve DI (red), best decision tree (green), and the best ANN (blue). This optimization was done with a random subset of 9 of the 25 benchmark proteins due to computational limitations. Using known contacts leads to greater improvement, which plateaus at 2L and a maximum average improvement with a minimum separation of 12 of 2.90Å. Naïve DI, and the best decision tree peak for L-fractions of L/2 and 1L at a minimum separation of 6 and 12 respectively (1.81Å for both). Finally, the best ANN peaks for the maximum L-fraction of 3L and a minimum separation of 12 with a maximum average improvement of 2.05Å. (DOCX) [file pone.0177866.s002.docx]

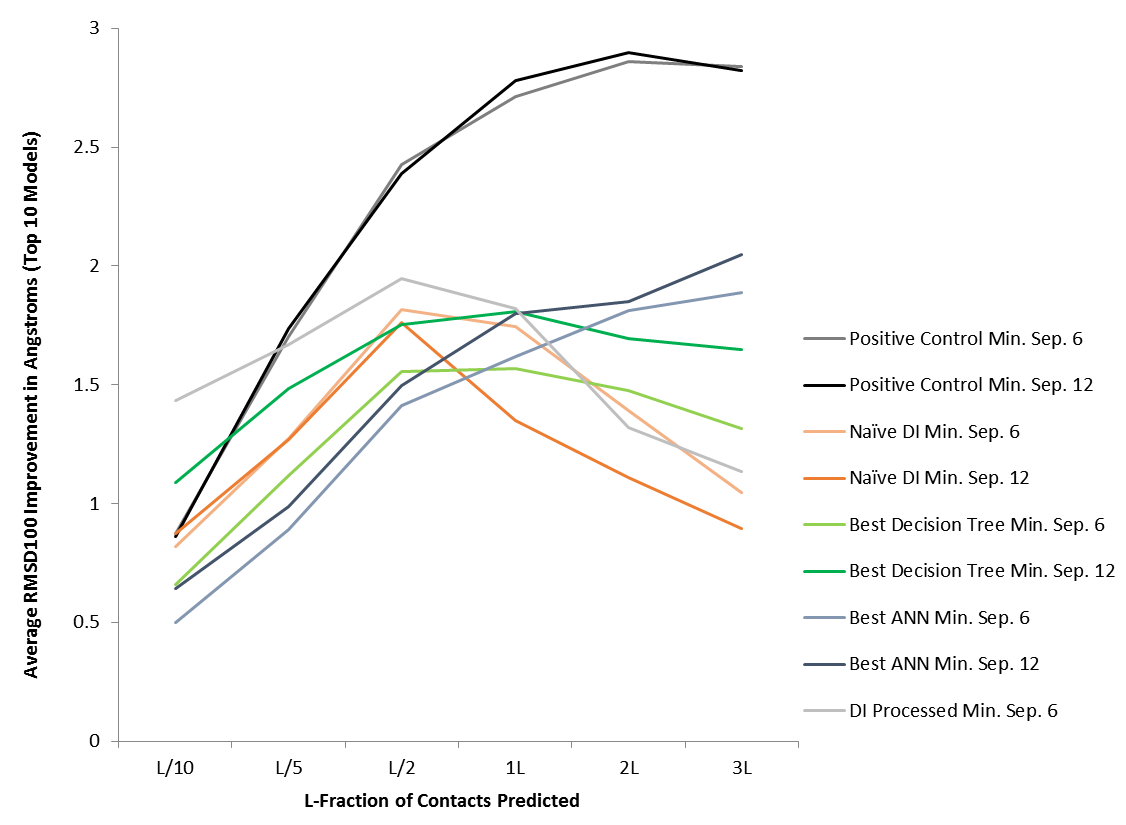


S2 Fig. L-Fraction Optimization for Structure Prediction Using Contacts from the Positive Control, Naïve Direct Information, Best Decision Tree, and Best ANN, Related to Table 3.

This is the average RMSD100 improvement for the top 10 models across the L-fractions examined with a minimum separation of 6 and 12 (light and dark colors respectively) for the positive control (black), naïve DI (red), best decision tree (green), and the best ANN (blue). This optimization was done with a random subset of 9 of the 25 benchmark proteins due to computational limitations. Using known contacts leads to greater improvement, which plateaus at 2L and a maximum average improvement with a minimum separation of 12 of 2.90Å. Naïve DI, and the best decision tree peak for L-fractions of L/2 and 1L at a minimum separation of 6 and 12 respectively (1.81Å for both). Finally, the best ANN peaks for the maximum L-fraction of 3L and a minimum separation of 12 with a maximum average improvement of 2.05Å.
