# Supplementary material for: Membrane protein contact and structure prediction using co-evolution in conjunction with machine learning
Source: PLoS One. 2017 May 24;12(5):e0177866. doi: 10.1371/journal.pone.0177866 (PMC5443516; doi:10.1371/journal.pone.0177866)
Supplement: S3 Fig — In addition to the input sensitivity iteration method used with decision trees, we also attempted a descriptor optimization that determines which descriptors are most useful by analyzing the weights of the ANN models trained. We graphed the results of a 29 round optimization for both the AUC and the integral of the positive predictive value across the fraction predicted positive from 0.01% to 0.55%. As we remove descriptors, the AUC slowly trends upwards. There is a slight plateau once one reaches 203 descriptors. However, the positive predictive value integral is largely steady within a range until one reaches 146 descriptors. This is the highest point, followed by several higher but declining values as one approaches the final round of optimization. Given that the positive predictive value integral is more representative of the top L contact predictions desired for protein fold prediction, we used the top 146 descriptors for final contact prediction (round 23). (DOCX) [file pone.0177866.s003.docx]

##
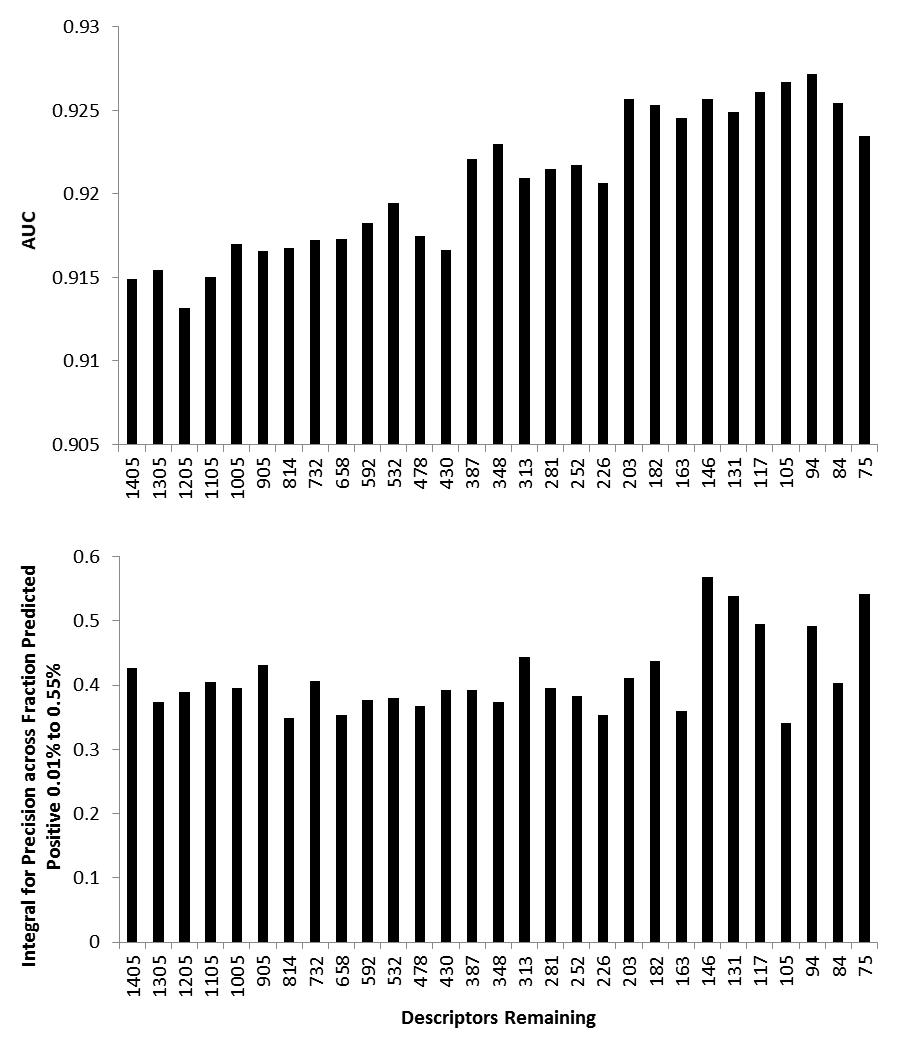


S3 Fig. AUC and Integral for Precision across Fraction Predicted Positive After All 29 Rounds of ANN Weights-Based Optimization, Related to Fig 1.

In addition to the input sensitivity iteration method used with decision trees, we also attempted a descriptor optimization that determines which descriptors are most useful by analyzing the weights of the ANN models trained. We graphed the results of a 29 round optimization for both the AUC and the integral of the positive predictive value across the fraction predicted positive from 0.01% to 0.55%. As we remove descriptors, the AUC slowly trends upwards. There is a slight plateau once one reaches 203 descriptors. However, the positive predictive value integral is largely steady within a range until one reaches 146 descriptors. This is the highest point, followed by several higher but declining values as one approaches the final round of optimization. Given that the positive predictive value integral is more representative of the top L contact predictions desired for protein fold prediction, we used the top 146 descriptors for final contact prediction (round 23).
