# Supplementary material for: Membrane protein contact and structure prediction using co-evolution in conjunction with machine learning
Source: PLoS One. 2017 May 24;12(5):e0177866. doi: 10.1371/journal.pone.0177866 (PMC5443516; doi:10.1371/journal.pone.0177866)
Supplement: S4 Fig — This is an example of the top L/10 predicted contacts for 3GD8A (left) showing both the distribution across the protein structure and the potential accuracy of the top DI pairs for contact prediction. The accuracy is approximately 91%. For this set, only two pairs are incorrect at 9.2Å and 18.1Å. The latter is a predicted contact between flexible loop regions. 3MKTA (middle) shows the distribution across the protein structure and is an example of the medium accuracy possible for the top DI pairs. Accuracy for this protein is 56.5%. Few pairs are incorrect, and most of them are within the 8-11Å range. Many of the incorrect pairs are still “near-contacts”. 2RH1A (right) shows one of the few examples of low accuracy in the absence of topology prediction assistance. Blue lines connect correct contact pairs (within the 8Å threshold). Yellow lines connect incorrect pairs that are “near-contacts” (between 8Å and 12Å). Red lines connect incorrect pairs. Accuracy for top L/10 is approximately 18.9%. (DOCX) [file pone.0177866.s004.docx]

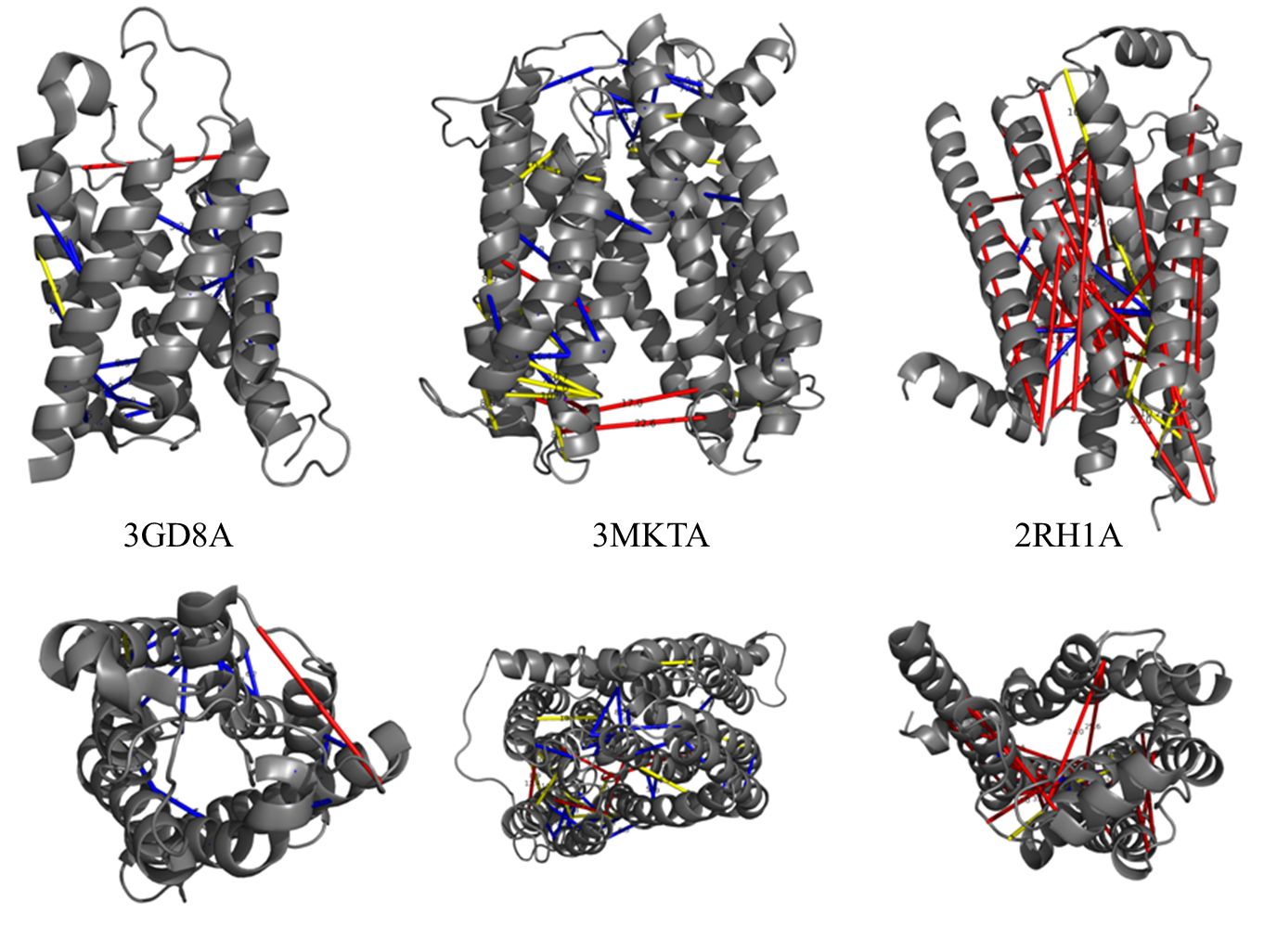


S4 Fig. High, Medium, and Low Accuracy Top L/10 Visualized Contacts for 3GD8A, 3MKTA, and 2RH1A respectively (DI-only Filtered), Related to Table 2.

This is an example of the top L/10 predicted contacts for 3GD8A (left) showing both the distribution across the protein structure and the potential accuracy of the top DI pairs for contact prediction. The accuracy is approximately 91%. For this set, only two pairs are incorrect at 9.2Å and 18.1Å. The latter is a predicted contact between flexible loop regions. 3MKTA (middle) shows the distribution across the protein structure and is an example of the medium accuracy possible for the top DI pairs. Accuracy for this protein is 56.5%. Few pairs are incorrect, and most of them are within the 8-11Å range. Many of the incorrect pairs are still “near-contacts”. 2RH1A (right) shows one of the few examples of low accuracy in the absence of topology prediction assistance. Blue lines connect correct contact pairs (within the 8Å threshold). Yellow lines connect incorrect pairs that are “near-contacts” (between 8Å and 12Å). Red lines connect incorrect pairs. Accuracy for top L/10 is approximately 18.9%.
