# Supplementary material for: Membrane protein contact and structure prediction using co-evolution in conjunction with machine learning
Source: PLoS One. 2017 May 24;12(5):e0177866. doi: 10.1371/journal.pone.0177866 (PMC5443516; doi:10.1371/journal.pone.0177866)
Supplement: S5 Fig — To determine the optimal difference threshold for filtering contacts to maximize accuracy we evaluated thresholds from zero to 0.95 for all top L-fractions and minimum separations of 6 and 12. Accuracy decreases initially for overly stringent cutoffs (near zero), which eliminate too many possible contacts for most sets of parameters. Values begin improving for most L-fractions and minimum separation values beginning around a maximum predicted transmembrane separation of 0.2. This improvement is optimal around 0.35 across L-fractions and as such, the filtering threshold was set to a maximum predicted vertical separation of 0.35. As the threshold increases beyond 0.35, the beneficial effects of topology prediction decrease slowly towards zero. The top 1L contact predictions decreases beyond zero during this latter range likely due to a clustering of contacts with inaccurate transmembrane separation values of 1.0 due to incorrect topology predictions in the range between the top L/2 and L contacts. (DOCX) [file pone.0177866.s005.docx]

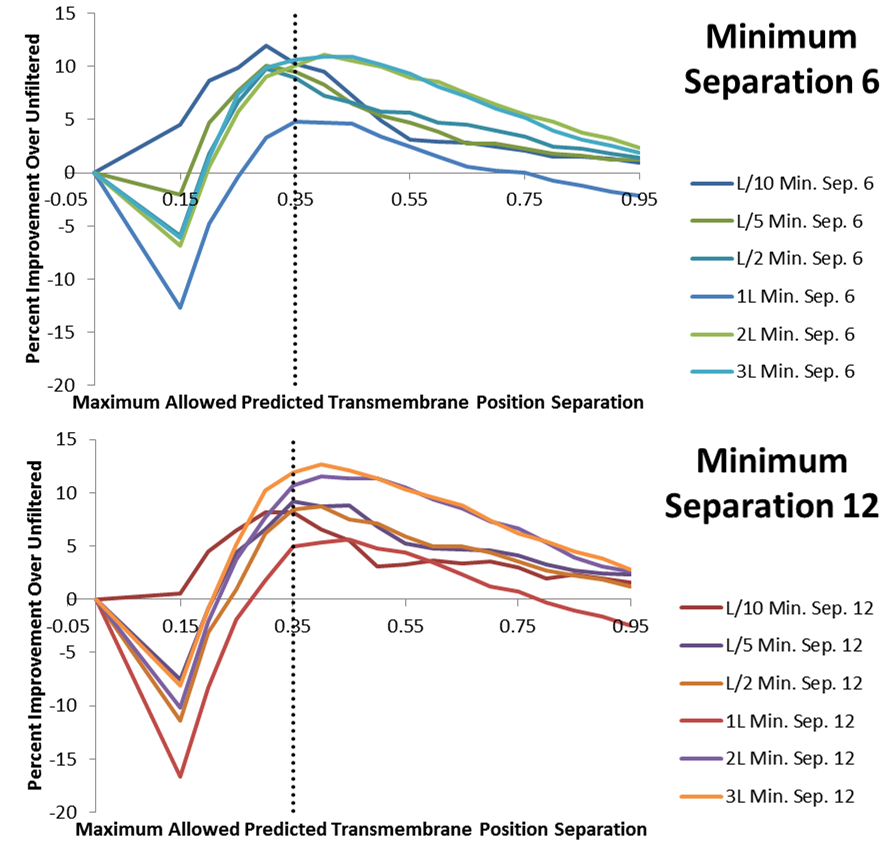


S5 Fig. Topology-based Filtering Threshold Selection, Related to Fig 2.

To determine the optimal difference threshold for filtering contacts to maximize accuracy we evaluated thresholds from zero to 0.95 for all top L-fractions and minimum separations of 6 and 12. Accuracy decreases initially for overly stringent cutoffs (near zero), which eliminate too many possible contacts for most sets of parameters. Values begin improving for most L-fractions and minimum separation values beginning around a maximum predicted transmembrane separation of 0.2. This improvement is optimal around 0.35 across L-fractions and as such, the filtering threshold was set to a maximum predicted vertical separation of 0.35. As the threshold increases beyond 0.35, the beneficial effects of topology prediction decrease slowly towards zero. The top 1L contact predictions decreases beyond zero during this latter range likely due to a clustering of contacts with inaccurate transmembrane separation values of 1.0 due to incorrect topology predictions in the range between the top L/2 and L contacts.
