# Supplementary material for: Membrane protein contact and structure prediction using co-evolution in conjunction with machine learning
Source: PLoS One. 2017 May 24;12(5):e0177866. doi: 10.1371/journal.pone.0177866 (PMC5443516; doi:10.1371/journal.pone.0177866)
Supplement: S6 Fig — The plot shows the relative benefit of using predictions from the best model, the optimized ANN at 3L minimum separation of 12, in comparison to folding without any contact restraints. Average RMSD100 for the top 10 models in each run are plotted such that the result from the negative control set is given on the x-axis and the set using the best predictions is on the y-axis. Equal performance is depicted via the dotted x = y line and any point below this diagonal is improved by inclusion of our predicted restraints. All 25 points are below the diagonal showing consistent improvement across the benchmark set. (DOCX) [file pone.0177866.s006.docx]

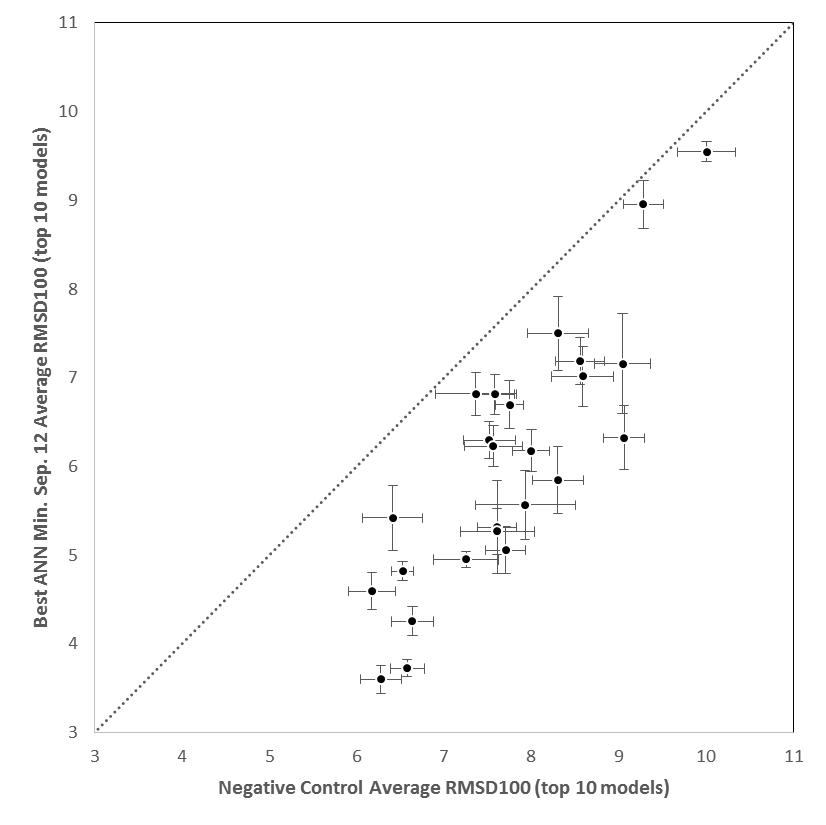


S6 Fig. RMSD-RMSD Comparison of the Top 10 Models from Runs with Contact Restraints from the Best Model (ANN) and without Any Contact Restraints, Related to Fig 6.

The plot shows the relative benefit of using predictions from the best model, the optimized ANN at 3L minimum separation of 12, in comparison to folding without any contact restraints. Average RMSD100 for the top 10 models in each run are plotted such that the result from the negative control set is given on the x-axis and the set using the best predictions is on the y-axis. Equal performance is depicted via the dotted x=y line and any point below this diagonal is improved by inclusion of our predicted restraints. All 25 points are below the diagonal showing consistent improvement across the benchmark set.
