# Supplementary material for: Membrane protein contact and structure prediction using co-evolution in conjunction with machine learning
Source: PLoS One. 2017 May 24;12(5):e0177866. doi: 10.1371/journal.pone.0177866 (PMC5443516; doi:10.1371/journal.pone.0177866)
Supplement: S7 Fig — We have included a comparison of the direct information values, running accuracy, and confidence weighting for 3MKTA, an average example of good contact prediction using direct information, and 1OCCA, a poorly performing example from an early direct information based method. The distribution and magnitude of the confidence weighting is relatively similar, while the direct information values are much larger and decrease more slowly for 3MKTA. The point where confidence weighting crosses the 1.0 threshold—distinguishes between the set of contacts weighted more and less heavily by confidence scoring. The accuracy for 3MKTA is much higher initially and stays around 30% across the entire set of the top L contact predictions. 1OCCA’s accuracy drops off much more precipitously and quickly approaches 0%. Thus, the confidence based score increases the weighting for many contacts that are in a range at or below 10% accuracy. (DOCX) [file pone.0177866.s007.docx]

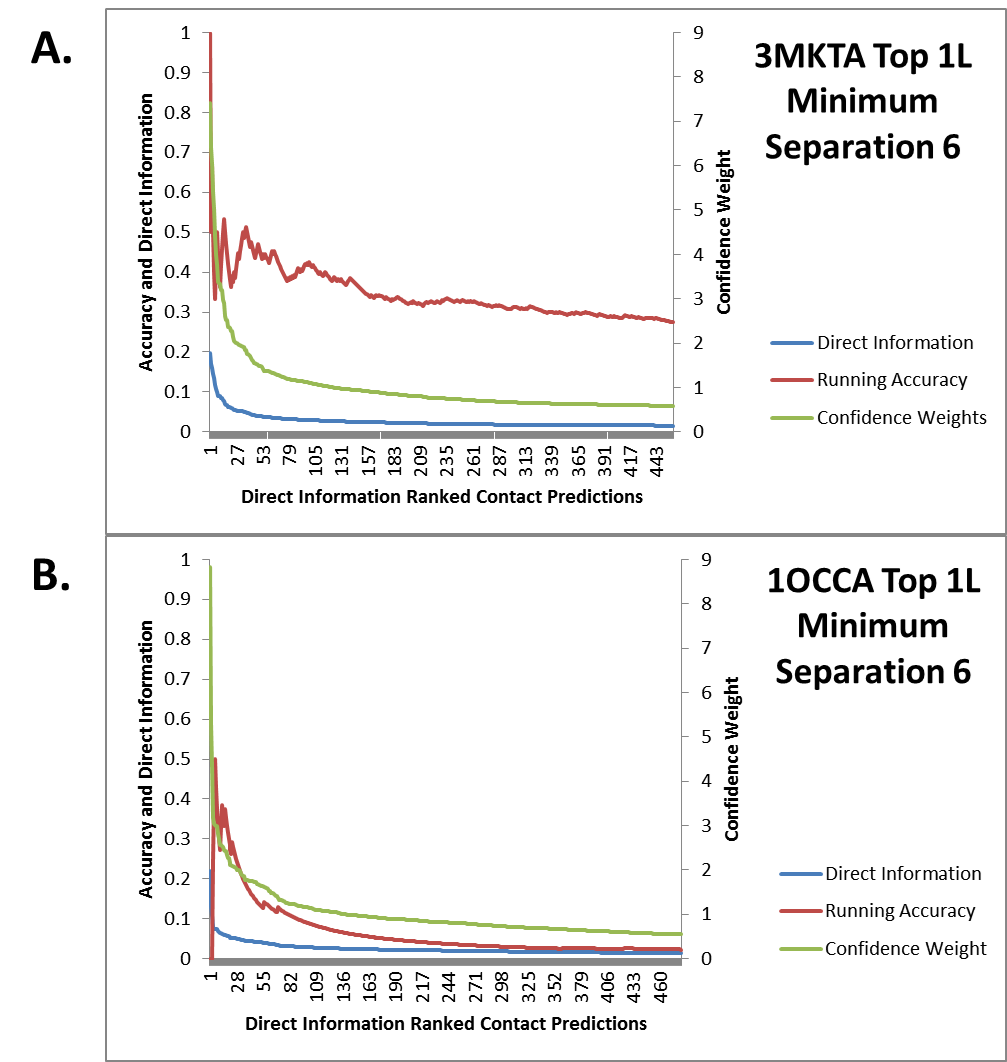


S7 Fig. Comparison of Direct Information, Running Accuracy, and Confidence Weights across the Top 1L Contacts for 3MKTA and 1OCCA, Related to Experimental Procedures.

We have included a comparison of the direct information values, running accuracy, and confidence weighting for 3MKTA, an average example of good contact prediction using direct information, and 1OCCA, a poorly performing example from an early direct information based method. The distribution and magnitude of the confidence weighting is relatively similar, while the direct information values are much larger and decrease more slowly for 3MKTA. The point where confidence weighting crosses the 1.0 threshold – distinguishes between the set of contacts weighted more and less heavily by confidence scoring. The accuracy for 3MKTA is much higher initially and stays around 30% across the entire set of the top L contact predictions. 1OCCA’s accuracy drops off much more precipitously and quickly approaches 0%. Thus, the confidence based score increases the weighting for many contacts that are in a range at or below 10% accuracy.
