# Supplementary material for: Membrane protein contact and structure prediction using co-evolution in conjunction with machine learning
Source: PLoS One. 2017 May 24;12(5):e0177866. doi: 10.1371/journal.pone.0177866 (PMC5443516; doi:10.1371/journal.pone.0177866)
Supplement: S1 File — This file includes supplementary information and explanations. (PDF) [file pone.0177866.s008.pdf]

Supplemental Information

Supplemental Experimental Procedures

MSA Coverage

Sequence coverage is a proxy for determining whether functional pressures were similar across all sequences included in the MSA. Evolutionary pressures result in the detected DI correlations essential to accurate contact prediction. A large amount of sequence diversity is necessary to capture sufficient mutations to accurately calculate DI. However, diversity to the point of a new functional role for a protein introduces different selective pressures, which confound DI calculation. An ideal DI calculation would isolate evolutionary coupling produced by the selective pressures associated with the overall functions of a single protein. The results seem relatively robust to the exact coverage value used, but generally, it seems that coverage in the range of 70% indicates a high probability of preserved functional pressures.

Definition of long-range contacts

A minimum separation of six residues removes the vast majority of trivial contacts. Such contacts are of especially low utility to BCL::Fold, which uses idealized secondary structures during folding, as contacts within an SSE are unlikely to alter folding predictions. A minimum separation of twelve residues is the traditional cutoff for "long-range" contact restraints. Increasing from six to twelve residues of separation decreases contacts by only between 6.3% and 22.3%, or 10% on average (data not shown). The topology of α-helical MPs and the high SSE content are such that pairs with a minimum

separation between six and twelve are unlikely to be in contact. Such pairs are often within and thus “held” apart by a rigid SSE. The only exception are sequential SSEs separated by a very short loop whose close proximity is already guaranteed by the loop potential within BCL::Fold. Therefore most contacts of interest for BCL::Fold have a sequence separation of at least twelve residues.

Maximum Number of Contacts in MPs

One should also note that for minimum separations of six and twelve the maximum number of possible contacts is on average 1.6L and 1.5L, respectively (data not shown). Thus, for a perfect contact prediction method taking any L-fraction beyond this range would be counter-productive as it would very likely extend beyond the set of possible contacts. However, there are two scenarios that might make usage of more than 1.5L contacts valuable: 1) the contact prediction method is imperfect and including more predicted contacts will capture more of the true positives and 2) often false positives of contact prediction methods are enriched for ‘near-contacts’, i.e. distances that are outside of the traditionally strict 8Å contact cutoff but still within a larger cutoff of 12-16Å. Therefore, it might be beneficial to include more than 1.5L contacts for structure prediction is a scoring function can be derived tolerates a fraction of the predicted contacts being not fulfilled by the model and gives some bonus for near-contacts.

Descriptor Optimization

The number of descriptors balloons to 1,505 when all the categories are used with all permutations (windows, aggregations of windows, different MSA parameters, applied to each property etc.). To optimize performance, it was necessary to reduce the number of descriptors significantly. Many were not beneficial for contact prediction and added noise and increased computational times. Ideally, one would use backwards elimination or forward feature selection, however due to computational and time constraints it was necessary to filter descriptors using other methods. We scored descriptors using information gain and F-score to determine their individual potential for contact prediction. F-score rapidly drops off from a maximum of 0.966 for predicted transmembrane separation to nearly zero by the 217th descriptor (data not shown). We used the descriptor ranking to train DT models using the top N descriptors. We evaluated performance with AUC initially. The smallest number of N descriptors was 10 and increased in increments of 50 descriptors to cover all descriptors. At each threshold we perform a fivefold cross validation and average the predictions across all generated models. During descriptor and parameter optimization we allowed data points from a single protein to span across training and monitoring or monitoring and independent datasets. However, at no point was any data point included within multiple datasets. The final DT was of simple structure with single splits per node and was restricted to a minimum of 10 examples per terminal node. The input was the top 30 descriptors identified as per the described procedure above (S2 Table).

The ANN utilized a single hidden layer with eight nodes and a single input and output node. We optimized the set of descriptors for ANNs using an iterative method that examines the weights between nodes with ANNs trained on the descriptors to be

optimized. Jeffrey Mendenhall from the Meiler Lab developed the method as part of the BCL. At its core, the method utilizes the weights between the nodes of an ANN to calculate an approximate derivative for each descriptor. For the weight matrix between layer x and y (M_{xy}), this method computes the product of the transpose of M_{xy} for each model given. The result of that product of matrices is the approximate partial derivative of the result dependent on the feature in question. This method then scores the descriptors using two previously implemented statistical measures. The first evaluates the consistency, whether the descriptor tends to increase or decrease the likelihood of a contact across all models given. The second is the average (pseudo) derivative squared. Each is then rescaled between 0 and 0.5, summed, and squared. Descriptors that are not generally useful should show little consistency and have small weights, so the outcome of this measure will range between 0 for non-general descriptors, and 1 for descriptors that are broadly useful. We have calculated the AUC and the integral of the precision from the previously specified range for each set of models and have graphed the results across all 29 iterations above. The AUC slowly trends upwards across the entire set of runs with a peak at the 27th iteration, which uses 94 descriptors. However, using the integral results, performance stays range-bound around 0.4 until the 23rd iteration at which point there is a jump up to approximately 0.569 followed by a slow decline over the next two runs before returning to similar performance as the initial iterations interspersed with a few nearly equivalent runs. For final ANN training we selected this 23rd round as it had both the highest value as well as several subsequent iterations with promising results. These 146 descriptors were the inputs for the final ANN model.

Optimizing L-fraction and Minimum Separation

Before progressing to a final comparison across methods, we first determined the best minimum separation and L-fraction (on average) for each method. We have included the results of this separation and L-optimization in S1 Fig. For each method, we generated 1000 models for the same subset of 9 of the 25 benchmark proteins across all L-fractions examined for a minimum separation of 6 and 12. We then determined the 10 best models by RMSD100 for each set of conditions and calculated the difference between the averages for this top set compared to the 10 best models for the models generated without any contact information. We determined the average RMSD100 improvement across all nine proteins and linked these values across L-fractions in the line graph in S2 Fig.

The positive control, with a minimum separation of 6 and 12 (gray and black), plateaus at approximately 2L contacts. The maximum occurs at 2L with a minimum separation of 12. There is very little difference between the different separation parameters for the control, but the predicted contacts have much more variation between the two separations – especially for contact fractions greater than L/2. Naïve DI is especially similar between the two for contact fractions less than or equal to L/2. The difference in performance also seems anti-correlated with the increasing accuracy from naïve DI, to the best DTs, and finally to the best ANNs. In addition, there also appears to be a correlation between accuracy and the L-fraction that results in the best BCL::Fold performance. As accuracy increases across these three examples the best L-fraction also increases – L/2 to 1L and finally to 3L. Naïve DI is also the only method with a maximum resulting from a set on contacts with a minimum separation of six. This may indicate that the method’s accuracy drops off more rapidly as one attempts to predict increasing numbers of contacts.

Machine learning based methods appear to reduce this trend, which may be because they do not rely solely on DI to rank potential contact pairs. We also examined processed DI and it performed nearly identically to naïve DI, as such, we did not include it. Finally, it is also interesting that the results from the best ANN models do not peak but rather continue to improve across the entire set of L-fractions examined. This is similar to the pattern seen with known contacts, as a larger fraction of false positive does not accompany the additional information that can confound predictions. Thus, the similarity of the ANN based method to the L-optimization trend seen with known constraints further suggests that ANNs are most accurate as well as beneficial for protein structure prediction.

Supplemental Equations

$$NM(i,j) = \frac{\sum_{x=i-k}^{i+k} \sum_{y=j-k}^{j+k} \frac{\boldsymbol{DI(x,y)}}{\boldsymbol{2}}}{\left(\frac{\sum_{z=1}^{L} \boldsymbol{DI(z)}}{\boldsymbol{L}}\right)} \qquad \textbf{(1)}$$

S1 Equation. Normalized Mean Calculation for the Given Correlation Window Surrounding Position *i,j*

Normalized mean (NM) for *i,j* is determined by calculating the average DI for the symmetric matrix surrounding *i,j* and dividing that by the average DI across the entire protein sequence. *L* is the length of the protein sequence and *k* represents half the window size desired rounded down. Window size was set to nine for this study.

$$M_{eff} = \sum_{a=1}^{M} \frac{1}{m^a} \quad (2)$$

S2 Equation. Effective number of MSA independent sequences

The effective number of sequences is calculated as the sum across the number of sequences (M) for one divided by the number of sequences similar to the ath sequence (m^a)[17].

$$F_s = 2\frac{pr}{p+r} \quad (3)$$

S3 Equation. F-Score

A measure of test accuracy that incorporates both the precision (p) and the recall (r) to determine the F-score (F_S).

$$D_{KL}(P||Q) = \sum_i P(i)\, log\, \frac{P(i)}{Q(i)} \quad (4)$$

S4 Equation. Kullback-Leibler divergence

Also known as “information gain” the Kullback-Leibler divergence approximates the information lost when one attempts to approximate the discrete probability distribution P with Q. This can also be thought of as the relative entropy for P in comparison with Q.
