# Supplementary material for: Membrane protein contact and structure prediction using co-evolution in conjunction with machine learning
Source: PLoS One. 2017 May 24;12(5):e0177866. doi: 10.1371/journal.pone.0177866 (PMC5443516; doi:10.1371/journal.pone.0177866)
Supplement: S1 Table — The table above contains all the categories of descriptors initially analyzed. They are divided into the three broad categories. The first is global position descriptors—the location of each element of the pair i,j being predicted within the context of the sequence. The second category is the sequence descriptors—biochemical, BLAST, and predicted secondary structure information regarding the amino acids as well as aggregated descriptors (mean and standard deviation) calculated across the entire sequence for the given properties and BLAST data. Finally, the third lists coupling descriptors, which includes various aggregated descriptors such as the max, mean, sequence normalized mean, standard deviation, and sum across collections of the elemental correlation descriptors (by window or across e-value/filtering parameters). (DOCX) [file pone.0177866.s009.docx]

**S1 Table.** **Categories of Global, Sequence, and Direct Information (Correlation) Descriptors, Related to Experimental Procedures**.

| Global Position Descriptors | |
| --- | --- |
| Sequence IDs for Positions i and j | Distance from Beginning of Sequence to i |
| Distance from i to j | Distance from j to End of Sequence |
|  |  |
| Sequence Descriptors | |
| Properties | **Predicted Secondary Structure Descriptors** |
| Sterical Parameter | SSE Size |
| Polarizability | Position in SSE |
| Hydrophobicity | Distance from SSE Center |
| IsoelectricPoint | Predicted Position in Membrane |
| Volume | SSE Index Difference |
| Helix Probability  Strand Probability | Predicted Vertical Membrane Separation (Topology-Based) |
| Free Energy Helix | JUFO Helix/Strand/Coil Probabilities |
| Free Energy Coil  Transfer Free Energy Punta-Maritan 3D | JUFO9D Membrane Transition Solution Probabilities |
| Free Energy Core | JUFO9D Membrane and SSE Probabilities |
| Free Energy Transition  Free Energy Solution | OCTOPUS Membrane Transition Solution Probabilities |
| Free Energy Core Helix |  |
| Free Energy Transition Helix  Free Energy Solution Helix | **Whole Sequence Descriptors (Applied to All Properties and BLAST Descriptors)** |
| Free Energy Core Coil | Sequence Mean |
| Free Energy Transition Coil | Sequence Std. Deviation |
| Free Energy Solution Coil |  |
|  | **Window Types (Applied to All Properties)** |
|  | Window |
| BLAST Descriptors | Window Average |
| BLAST Profile  BLAST Conservation | Window Standard Deviation |
|  | Window with Properties Weighted by BLAST Log Probability |
|  | Sequence Mean across All Properies |
|  | Sequence Std. Deviation across All Properies |
|  |  |
| Direct Information Descriptors | |
| Correlation Groups  Filtered MSA Using Opt. E-values | **Window Types (Applied to All Correlation Groups)** |
| Unfiltered MSA Using Opt. E-values | Window Max |
| Across All Filtered MSA (1E-03, 1E-05, 1E-10, 1E-15, 1E-20, 1E-30, and 1E-40) | Window Mean  Window Normalized Mean |
| Across All Unfiltered MSA (1E-03, 1E-05, 1E-10, 1E-15, 1E-20, 1E-30, and 1E-40) | Window Std. Deviation  Window Sum |
| Across All Filtered and Unfiltered MSA (1E-03, 1E-05, 1E-10, 1E-15, 1E-20, 1E-30, and 1E-40) |  |
|  |  |
|  |  |
| MSA Statistics Descriptors |  |
| MSA Length |  |
| Effective MSA Depth (M_eff_) |  |
| MSA Depth (M) |  |
| Target Sequence Coverage |  |
|  |  |

The table above contains all the categories of descriptors initially analyzed. They are divided into the three broad categories. The first is global position descriptors - the location of each element of the pair i,j being predicted within the context of the sequence. The second category is the sequence descriptors – biochemical, BLAST, and predicted secondary structure information regarding the amino acids as well as aggregated descriptors (mean and standard deviation) calculated across the entire sequence for the given properties and BLAST data. Finally, the third lists coupling descriptors, which includes various aggregated descriptors such as the max, mean, sequence normalized mean, standard deviation, and sum across collections of the elemental correlation descriptors (by window or across e-value/filtering parameters).
