# Supplementary material for: Membrane protein contact and structure prediction using co-evolution in conjunction with machine learning
Source: PLoS One. 2017 May 24;12(5):e0177866. doi: 10.1371/journal.pone.0177866 (PMC5443516; doi:10.1371/journal.pone.0177866)
Supplement: S2 Table — This is the final set of 30 descriptors selected for use with DTs to predict long-range contacts. We determined this set using an iterative process whereby we scored the usefulness of descriptors for a given model type initially using F-score and then input sensitivity. We evaluated models using increasing subsets of the ranked descriptors. We then selected optimal thresholds and repeated the scoring and evaluation process until improvement plateaued or decreased. The top 30 descriptors are described above along with the type of descriptor and the input sensitivity at the last iteration. (DOCX) [file pone.0177866.s010.docx]

**S2 Table. Top 30 Descriptors Used for Best DT Model, Related to Fig 1.**

| Descriptor Name | Input Sensitivity Score | Type | Rank |
| --- | --- | --- | --- |
| Window Maximum Correlation Filtered [Optimized] | 0.02013 | Correlation | 1 |
| Amino Acid Sequence Separation | 0.01601 | Sequence | 2 |
| Predicted Transmembrane Separation | 0.01027 | Topology | 3 |
| Normalized Window Mean Correlation Unfiltered [Optimized] | 0.00604 | Correlation | 4 |
| Window Max Correlation Unfiltered [Optimized] | 0.00545 | Correlation | 5 |
| Sequence Mean Polarizability | 0.00298 | Sequence | 6 |
| Normalized Window Mean Correlation E-value 1E-10 Unfiltered MSA | 0.00193 | Correlation | 7 |
| Sequence Mean(Correlation(Filtered [Optimized] | 0.00179 | Correlation | 8 |
| Position *i* Sequence Position | 0.00172 | Sequence | 9 |
| Coverage for E-value 1E-10 Filtered MSA | 0.00127 | MSA Statistics | 10 |
| Probability (JUFO9D) Amino Acid j is a Transition Region Coil | 0.00121 | Sequence | 11 |
| Normalized Mean Window Correlation E-value 1E-40 Unfiltered MSA | 0.00119 | Correlation | 12 |
| Max Correlation All Filtered and Unfiltered MSA | 0.00118 | Correlation | 13 |
| Normalized Mean Correlation Filtered [Optimized] | 0.00074 | Correlation | 14 |
| Correlation Unfiltered [Optimized] Center Position | 0.00072 | Correlation | 15 |
| Normalized Window Mean Correlation E-value 1E-30 Unfiltered MSA | 0.00067 | Correlation | 16 |
| Sequence Length (L) | 0.00065 | Sequence | 17 |
| Correlation Filtered [Optimized] Center Position | 0.00049 | Correlation | 18 |
| Amino Acid Sequence ID Difference | 0.00046 | Global Position | 19 |
| Distance to End of Sequence | 0.00040 | Global Position | 20 |
| Sequence Mean IsoelectricPoint | 0.00036 | Sequence | 21 |
| Meff for E-value 1E-03 Filtered MSA | 0.00035 | MSA Statistics | 22 |
| Window Average of Blast Log Weighted Free Energy Transition Coil with Triangular Weighting at Index 7 | 0.00034 | Sequence | 23 |
| Correlation E-value 1E-10 Unfiltered MSA | 0.00031 | Correlation | 24 |
| Mean Correlation All Filtered MSA | 0.00031 | Correlation | 25 |
| Probability (JUFO9D) Amino Acid *i* is in a Helix | 0.00030 | Sequence | 26 |
| Mean Correlation All Filtered and Unfiltered MSA | 0.00030 | Correlation | 27 |
| Normalized Window Mean Correlation E-value 1E-40 Filtered MSA | 0.00028 | Correlation | 28 |
| Correlation E-value 1E-03 Unfiltered MSA | 0.00027 | Correlation | 29 |
| Distance from Beginning of Sequence to Position i | 0.00026 | Global Position | 30 |

This is the final set of 30 descriptors selected for use with DTs to predict long-range contacts. We determined this set using an iterative process whereby we scored the usefulness of descriptors for a given model type initially using F-score and then input sensitivity. We evaluated models using increasing subsets of the ranked descriptors. We then selected optimal thresholds and repeated the scoring and evaluation process until improvement plateaued or decreased. The top 30 descriptors are described above along with the type of descriptor and the input sensitivity at the last iteration.
