# Supplementary material for: Membrane protein contact and structure prediction using co-evolution in conjunction with machine learning
Source: PLoS One. 2017 May 24;12(5):e0177866. doi: 10.1371/journal.pone.0177866 (PMC5443516; doi:10.1371/journal.pone.0177866)
Supplement: S3 Table — This table lists the final set of accuracies for the best model from each method category (naïve DI, processed filtered DI, DTs, ANNs) with all optimal L-fractions for the given methods as well as L/10 at a minimum separation of 12 to show some of the highest precision prediction sets. All include the length (L), number of transmembrane helices (TMhelix), the effective alignment depth (Meff), and target sequence coverage (Cov) matched to each PDBID for comparison. Additionally, the best accuracy for each PDBID and L-fraction is bolded. DTs outperform all other methods at L/10 and a minimum separation of 12 (45.0%) and at L/2 and a minimum separation of 6 (29.4%). ANNs outperform all other methods at 1L and a minimum separation 12 (24.0%) and at 3L and a minimum separation of 12 (15.3%). (DOCX) [file pone.0177866.s011.docx]

**S3 Table. Table of Aggregated Contact Prediction Accuracies from Best Methods Across Categories and Optimal L-fractions for Each Included Method.**

|  | **DI Filtered MSA Stats** | | | | | | | | **DI Naïve Accuracy (%)** | | | | | | | | **DI Processed Filt Accuracy (%)** | | | | | | | |
| --- | --- | --- | --- | --- | --- | --- | --- | --- | --- | --- | --- | --- | --- | --- | --- | --- | --- | --- | --- | --- | --- | --- | --- | --- |
| **PDBID** | **L** | | **TM_helix_** | | **M_eff_** | | **Cov** | | **L/10 ms 12** | | **L/2 ms 6** | | **1L ms 12** | | **3L ms 12** | | **L/10 ms 12** | | **L/2 ms 6** | | **1L ms 12** | | **3L ms 12** | |
| **3NCYA** | 422 | | 12 | | 1205 | | 0.891 | | 42.2 | | 25.1 | | 18.7 | | 9.3 | | **44.4** | | 26.5 | | **20.9** | | 10.0 | |
| **2RH1A** | 442 | | 8 | | 451 | | 0.652 | | 18.9 | | 9.7 | | 6.7 | | 3.6 | | **21.6** | | 14.0 | | 8.9 | | 5.1 | |
| **1OKCA** | 292 | | 6 | | 1043 | | 0.89 | | **43.3** | | **34.2** | | 19.5 | | 10.2 | | 36.7 | | **34.2** | | **22.2** | | 10.9 | |
| **1XQFA** | 362 | | 11 | | 1021 | | 0.961 | | 28.6 | | 23.9 | | 15.3 | | 7.3 | | 52.4 | | 28.2 | | 17.7 | | 8.3 | |
| **3GD8A** | 223 | | 7 | | 1062 | | 0.964 | | 90.9 | | **59.8** | | 39.5 | | 18.5 | | 90.9 | | 53.6 | | 36.3 | | 18.4 | |
| **1L7VA** | 324 | | 10 | | 1045 | | 0.914 | | 0.0 | | 1.8 | | 1.2 | | 1.4 | | 0.0 | | 3.1 | | 3.1 | | 2.3 | |
| **3MKTA** | 460 | | 12 | | 1068 | | 0.917 | | **52.2** | | 32.2 | | 18.7 | | 8.7 | | 50.0 | | 32.6 | | 20.9 | | 10.7 | |
| **3RKON** | 473 | | 14 | | 1722 | | 0.831 | | 44.9 | | 18.9 | | 13.4 | | 7.2 | | 49.0 | | 22.2 | | 15.9 | | 9.4 | |
| **1OCCA** | 514 | | 12 | | 754 | | 0.979 | | 70.8 | | 42.7 | | 31.1 | | 14.7 | | **77.1** | | **52.7** | | 34.4 | | 17.6 | |
| **1OCCC** | 261 | | 6 | | 521 | | 0.958 | | 65.4 | | 29.0 | | 19.2 | | 8.7 | | 61.5 | | 29.8 | | 18.8 | | 7.9 | |
| **1PP9C** | 379 | | 8 | | 581 | | 0.921 | | 73.7 | | 29.0 | | 17.4 | | 7.9 | | **79.0** | | 32.1 | | 21.1 | | 9.5 | |
| **3H90A** | 283 | | 6 | | 1039 | | 0.968 | | 45.0 | | 31.7 | | 19.9 | | 10.0 | | 50.0 | | 29.7 | | 16.4 | | 9.1 | |
| **3B45A** | 180 | | 6 | | 1073 | | 0.867 | | 66.7 | | 41.1 | | 28.3 | | 12.4 | | **77.8** | | 44.4 | | 27.2 | | 13.2 | |
| **1PW4A** | 434 | | 12 | | 1604 | | 0.878 | | 8.9 | | 11.1 | | 7.8 | | 4.5 | | 13.3 | | 10.2 | | 9.5 | | 5.3 | |
| **3DHWA** | 203 | | 5 | | 1065 | | 0.877 | | **54.6** | | 30.3 | | 23.0 | | 10.6 | | 50.0 | | 31.2 | | 24.4 | | 11.4 | |
| **1YMGA** | 233 | | 7 | | 1010 | | 0.901 | | 15.4 | | 12.1 | | 9.9 | | 5.3 | | 15.4 | | 12.9 | | 9.1 | | 5.6 | |
| **3B60A** | 572 | | 6 | | 1568 | | 0.881 | | **42.4** | | 20.9 | | 16.0 | | 8.3 | | **42.4** | | **27.6** | | **16.9** | | **8.7** | |
| **2A65A** | 510 | | 12 | | 1043 | | 0.825 | | 59.6 | | 31.9 | | 22.2 | | 11.2 | | 55.8 | | 35.0 | | 25.8 | | 13.4 | |
| **1HZXA** | 340 | | 7 | | 1151 | | 0.803 | | 31.3 | | 23.3 | | 17.7 | | 8.1 | | 43.8 | | **29.6** | | 21.1 | | 9.5 | |
| **3PJZA** | 468 | | 12 | | 923 | | 0.793 | | 6.1 | | 6.1 | | 3.4 | | 2.0 | | **10.2** | | **7.3** | | 3.2 | | 2.2 | |
| **2XUTA** | 456 | | 14 | | 1040 | | 0.706 | | 32.7 | | 14.1 | | 8.6 | | 5.0 | | 38.5 | | 17.6 | | 11.6 | | 6.4 | |
| **3ZUXA** | 308 | | 10 | | 1005 | | 0.899 | | 66.7 | | 34.9 | | 23.2 | | 12.2 | | **69.7** | | 38.0 | | 26.2 | | 14.1 | |
| **2XQ2A** | 538 | | 15 | | 1380 | | 0.82 | | 0.0 | | 1.7 | | 1.0 | | 0.9 | | 1.7 | | 2.7 | | 1.2 | | 1.4 | |
| **3M71A** | 306 | | 10 | | 646 | | 0.971 | | 6.5 | | 10.8 | | 8.0 | | 6.2 | | 9.7 | | 16.6 | | 13.4 | | 8.1 | |
| **3QE7A** | 407 | | 14 | | 1355 | | 0.818 | | 60.5 | | 32.1 | | 22.8 | | 11.9 | | **69.8** | | 31.2 | | 23.5 | | 12.4 | |
| **Avg** | 376 | | 9.68 | | 1055 | | 0.875 | | 41.1 | | 24.3 | | 16.5 | | 8.2 | | 44.4 | | 26.5 | | 18.0 | | 9.2 | |
|  | | **DI Filtered MSA Stats** | | | | | | **Best DT Accuracy (%)** | | | | | | | | **Best ANN Accuracy (%)** | | | | | | | |  |
| **PDBID** | | **L** | | **TM_helix_** | **M_eff_** | **Cov** | | **L/10 ms 12** | | **L/2 ms 6** | | **1L ms 12** | | **3L ms 12** | | **L/10 ms 12** | | **L/2 ms 6** | | **1L ms 12** | | **3L ms 12** | |  |
| **3NCYA** | | 422 | | 12 | 1205 | 0.891 | | **42.2** | | **32.7** | | **20.9** | | **13.8** | | 8.9 | | 15.7 | | 15.1 | | 13.5 | |  |
| **2RH1A** | | 442 | | 8 | 451 | 0.652 | | 37.8 | | 19.9 | | 13.4 | | 8.7 | | **56.8** | | **28.0** | | **23.7** | | **13.4** | |  |
| **1OKCA** | | 292 | | 6 | 1043 | 0.89 | | 30.0 | | 32.9 | | 19.5 | | 11.0 | | 33.3 | | 25.5 | | 18.5 | | **15.2** | |  |
| **1XQFA** | | 362 | | 11 | 1021 | 0.961 | | **57.1** | | **31.1** | | **21.5** | | 12.7 | | 35.7 | | 25.4 | | 19.6 | | **14.0** | |  |
| **3GD8A** | | 223 | | 7 | 1062 | 0.964 | | 63.6 | | 49.1 | | 36.3 | | 20.9 | | **95.5** | | 58.9 | | **48.4** | | **27.7** | |  |
| **1L7VA** | | 324 | | 10 | 1045 | 0.914 | | **15.2** | | 12.3 | | 11.4 | | 8.8 | | 12.1 | | **15.3** | | **13.5** | | **11.2** | |  |
| **3MKTA** | | 460 | | 12 | 1068 | 0.917 | | 39.1 | | 35.7 | | **29.1** | | 15.7 | | 39.1 | | **36.1** | | **29.1** | | **22.0** | |  |
| **3RKON** | | 473 | | 14 | 1722 | 0.831 | | **63.3** | | **38.3** | | 24.5 | | 13.1 | | 51.0 | | 32.1 | | **31.6** | | **19.2** | |  |
| **1OCCA** | | 514 | | 12 | 754 | 0.979 | | **77.1** | | **57.7** | | **36.1** | | 18.1 | | 72.9 | | 36.9 | | 30.5 | | **20.2** | |  |
| **1OCCC** | | 261 | | 6 | 521 | 0.958 | | 61.5 | | 35.9 | | 22.2 | | 12.3 | | **80.8** | | **41.2** | | **27.2** | | **13.7** | |  |
| **1PP9C** | | 379 | | 8 | 581 | 0.921 | | 76.3 | | **36.3** | | **23.2** | | 11.3 | | 34.2 | | 30.0 | | 21.9 | | 12.3 | |  |
| **3H90A** | | 283 | | 6 | 1039 | 0.968 | | 45.0 | | **37.6** | | 24.4 | | **16.9** | | **55.0** | | 35.6 | | **25.9** | | 16.6 | |  |
| **3B45A** | | 180 | | 6 | 1073 | 0.867 | | **77.8** | | **46.7** | | 28.3 | | 16.5 | | 50.0 | | 43.3 | | **35.6** | | **21.7** | |  |
| **1PW4A** | | 434 | | 12 | 1604 | 0.878 | | 8.9 | | 12.4 | | 15.5 | | 11.5 | | **33.3** | | **20.8** | | **17.1** | | **12.8** | |  |
| **3DHWA** | | 203 | | 5 | 1065 | 0.877 | | 40.9 | | **37.6** | | 28.1 | | 14.4 | | 45.5 | | 32.1 | | **28.6** | | **17.1** | |  |
| **1YMGA** | | 233 | | 7 | 1010 | 0.901 | | 15.4 | | 14.4 | | 11.0 | | 10.0 | | **38.5** | | **31.1** | | **26.2** | | **19.0** | |  |
| **3B60A** | | 572 | | 6 | 1568 | 0.881 | | 15.2 | | 4.3 | | 4.0 | | 3.2 | | 15.2 | | 4.9 | | 7.7 | | 6.3 | |  |
| **2A65A** | | 510 | | 12 | 1043 | 0.825 | | **67.3** | | **37.3** | | 22.5 | | 12.8 | | 63.5 | | 31.5 | | **26.8** | | **15.1** | |  |
| **1HZXA** | | 340 | | 7 | 1151 | 0.803 | | **65.6** | | 28.3 | | 24.0 | | 14.3 | | 56.3 | | **29.6** | | **31.6** | | **19.7** | |  |
| **3PJZA** | | 468 | | 12 | 923 | 0.793 | | **10.2** | | 6.9 | | 4.5 | | 2.6 | | 8.2 | | 6.1 | | **7.7** | | **6.0** | |  |
| **2XUTA** | | 456 | | 14 | 1040 | 0.706 | | **51.9** | | **32.4** | | **22.0** | | **12.2** | | 42.3 | | 27.5 | | 21.8 | | 11.6 | |  |
| **3ZUXA** | | 308 | | 10 | 1005 | 0.899 | | 66.7 | | **45.8** | | 32.5 | | 18.5 | | 66.7 | | 44.0 | | **36.1** | | **19.8** | |  |
| **2XQ2A** | | 538 | | 15 | 1380 | 0.82 | | 0.0 | | 4.7 | | 1.7 | | 2.1 | | **6.8** | | **7.1** | | **6.6** | | **4.8** | |  |
| **3M71A** | | 306 | | 10 | 646 | 0.971 | | 29.0 | | 12.7 | | 12.7 | | 10.9 | | **38.7** | | **29.3** | | **21.7** | | **14.4** | |  |
| **3QE7A** | | 407 | | 14 | 1355 | 0.818 | | 67.4 | | 32.6 | | 23.8 | | **16.5** | | 39.5 | | **33.0** | | **27.0** | | 15.1 | |  |
| **Avg** | | 376 | | 9.68 | 1055 | 0.875 | | **45.0** | | **29.4** | | 20.5 | | 12.3 | | 43.2 | | 28.8 | | **24.0** | | **15.3** | |  |

This table lists the final set of accuracies for the best model from each method category (naïve DI, processed filtered DI, DTs, ANNs) with all optimal L-fractions for the given methods as well as L/10 at a minimum separation of 12 to show some of the highest precision prediction sets. All include the length (*L*), number of transmembrane helices (*TM_helix_*), the effective alignment depth (*M_eff_*), and target sequence coverage (*Cov*) matched to each PDBID for comparison. Additionally, the best accuracy for each PDBID and L-fraction is bolded. DTs outperform all other methods at L/10 and a minimum separation of 12 (45.0%) and at L/2 and a minimum separation of 6 (29.4%). ANNs outperform all other methods at 1L and a minimum separation 12 (24.0%) and at 3L and a minimum separation of 12 (15.3%).
