# Supplementary material for: Membrane protein contact and structure prediction using co-evolution in conjunction with machine learning
Source: PLoS One. 2017 May 24;12(5):e0177866. doi: 10.1371/journal.pone.0177866 (PMC5443516; doi:10.1371/journal.pone.0177866)
Supplement: S4 Table — Comparison across methods using positive predictive value of underlying method (DCA), proposed methods (DT: decision trees, ANN: artificial neural networks) and three state-of-art methods (PSICOV, CCMpred and FreeContact) at three separation cut-offs (6+, 12+ and 24+ residues), as well as 4 inclusion thresholds (L/10, L/5, L/2, L, where L is the length of protein). All results are based on the same alignments. We would like to emphasize, that this method is based on a first-generation mean field DCA implementation. Current methods for evolutionary coupling analysis take advantage of known improvements in the field in form of better sequence reweighting, use of regularization instead of pseudocounts and inferring more appropriate statistical models (Potts models instead of Ising models of original DCA). To facilitate comparison, we have based all the predictions (both by our method and the others) on the same alignments. We posit, that the methods we propose should be successfully applicable to the coupling inference methods of newer generations. (DOCX) [file pone.0177866.s012.docx]

**S4 Table. Mean positive predictive value of underlying method (DCA), proposed methods (DT: decision trees, ANN: artificial neural networks) and three state-of-art methods (PSICOV, CCMpred and FreeContact)**

|  |  | **DCA** | **DT** | | **ANN** | **PSICOV** | **FreeContact** | **CCMpred** |
| --- | --- | --- | --- | --- | --- | --- | --- | --- |
| L/10 | 6+ | 0.42 | | 0.46 | 0.40 | 0.60 | 0.66 | 0.71 |
|  | 12+ | 0.42 | | 0.46 | 0.44 | 0.58 | 0.67 | 0.69 |
|  | 24+ | 0.39 | | 0.44 | 0.41 | 0.55 | 0.65 | 0.67 |
| L/5 | 6+ | 0.36 | | 0.41 | 0.38 | 0.52 | 0.58 | 0.63 |
|  | 12+ | 0.35 | | 0.41 | 0.39 | 0.50 | 0.58 | 0.63 |
|  | 24+ | 0.33 | | 0.40 | 0.37 | 0.47 | 0.55 | 0.60 |
| L/2 | 6+ | 0.26 | | 0.32 | 0.32 | 0.36 | 0.44 | 0.48 |
|  | 12+ | 0.25 | | 0.31 | 0.32 | 0.34 | 0.44 | 0.46 |
|  | 24+ | 0.23 | | 0.29 | 0.30 | 0.31 | 0.41 | 0.42 |
| L | 6+ | 0.19 | | 0.25 | 0.26 | 0.25 | 0.32 | 0.34 |
|  | 12+ | 0.17 | | 0.23 | 0.26 | 0.23 | 0.31 | 0.32 |
|  | 24+ | 0.16 | | 0.22 | 0.26 | 0.21 | 0.28 | 0.28 |

Comparison across methods using positive predictive value of underlying method (DCA), proposed methods (DT: decision trees, ANN: artificial neural networks) and three state-of-art methods (PSICOV, CCMpred and FreeContact) at three separation cut-offs (6+, 12+ and 24+ residues), as well as 4 inclusion thresholds (L/10, L/5, L/2, L, where L is the length of protein). All results are based on the same alignments. We would like to emphasize, that this method is based on a first-generation mean field DCA implementation. Current methods for evolutionary coupling analysis take advantage of known improvements in the field in form of better sequence reweighting, use of regularization instead of pseudocounts and inferring more appropriate statistical models (Potts models instead of Ising models of original DCA). To facilitate comparison, we have based all the predictions (both by our method and the others) on the same alignments. We posit, that the methods we propose should be successfully applicable to the coupling inference methods of newer generations.
