# Supplementary material for: Membrane protein contact and structure prediction using co-evolution in conjunction with machine learning
Source: PLoS One. 2017 May 24;12(5):e0177866. doi: 10.1371/journal.pone.0177866 (PMC5443516; doi:10.1371/journal.pone.0177866)
Supplement: S5 Table — Here we have replicated Table 3 of main manuscript containing the same results, but with GDT-TS and TMscore as similarity metrics. While not as wide-spread as TM-score or GDT-TS, RMSD100 is also a metric for length-independent comparison of structural similarity and enables comparison to prior work using BCL::Fold. (DOCX) [file pone.0177866.s013.docx]

**S5 Table. Folding results using alternate similarity metrics GDT-TS and TMscore**

GDT-TS results

|  | **DI Filtered MSA Stats** | | | | **Negative Best RMSD100** | | | **Pos. Control 2L ms 12** | | |
| --- | --- | --- | --- | --- | --- | --- | --- | --- | --- | --- |
| **PDBID** | ***L*** | ***TM_helix_*** | ***M_eff_*** | ***Cov*** | **Best** | **Top 10 Avg** | **S.D.** | **Best** | **Top 10 Avg** | **S.D.** |
| **3NCYA** | **422** | 12 | 1205 | 0.89 | 0.2083 | 0.1766 | 0.0114 | 0.5186 | 0.4891 | 0.0784 |
| **2RH1A** | **442** | 8 | 451 | 0.65 | 0.289 | 0.2528 | 0.019 | 0.4832 | 0.4524 | 0.0631 |
| **1OKCA** | **292** | 6 | 1043 | 0.89 | 0.2562 | 0.2380 | 0.0168 | 0.4625 | 0.4393 | 0.0666 |
| **1XQFA** | **362** | 11 | 1021 | 0.96 | 0.2155 | 0.2069 | 0.0161 | 0.5254 | 0.5149 | 0.0967 |
| **3GD8A** | **223** | 7 | 1062 | 0.96 | 0.3454 | 0.3165 | 0.0266 | 0.5761 | 0.5521 | 0.0801 |
| **1L7VA** | **324** | 10 | 1045 | 0.91 | 0.2728 | 0.2399 | 0.0173 | 0.5637 | 0.5427 | 0.0975 |
| **3MKTA** | **460** | 12 | 1068 | 0.92 | 0.1927 | 0.1778 | 0.012 | 0.4114 | 0.3696 | 0.0423 |
| **3RKON** | **473** | 14 | 1722 | 0.83 | 0.1755 | 0.1621 | 0.0114 | 0.4763 | 0.4298 | 0.0561 |
| **1OCCA** | **514** | 12 | 754 | 0.98 | 0.2361 | 0.2072 | 0.0166 | 0.4976 | 0.4844 | 0.0649 |
| **1OCCC** | **261** | 6 | 521 | 0.96 | 0.3777 | 0.3530 | 0.0279 | 0.6559 | 0.6310 | 0.0576 |
| **1PP9C** | **379** | 8 | 581 | 0.92 | 0.2438 | 0.2211 | 0.0158 | 0.4885 | 0.4614 | 0.0628 |
| **3H90A** | **283** | 6 | 1039 | 0.97 | 0.3289 | 0.3010 | 0.0204 | 0.5965 | 0.5402 | 0.0620 |
| **3B45A** | **180** | 6 | 1073 | 0.87 | 0.3542 | 0.3354 | 0.0265 | 0.5845 | 0.5361 | 0.0725 |
| **1PW4A** | **434** | 12 | 1604 | 0.88 | 0.1977 | 0.1906 | 0.0141 | 0.5054 | 0.4874 | 0.0678 |
| **3DHWA** | **203** | 5 | 1065 | 0.88 | 0.2827 | 0.2722 | 0.0174 | 0.4085 | 0.3999 | 0.0384 |
| **1YMGA** | **233** | 7 | 1010 | 0.9 | 0.3214 | 0.3068 | 0.0242 | 0.5526 | 0.5458 | 0.0820 |
| **3B60A** | **572** | 6 | 1568 | 0.88 | 0.2643 | 0.2441 | 0.0204 | 0.4355 | 0.3964 | 0.0413 |
| **2A65A** | **510** | 12 | 1043 | 0.83 | 0.1996 | 0.1600 | 0.0099 | 0.5061 | 0.4687 | 0.0801 |
| **1HZXA** | **340** | 7 | 1151 | 0.8 | 0.2031 | 0.1964 | 0.0157 | 0.2000 | 0.1835 | 0.0102 |
| **3PJZA** | **468** | 12 | 923 | 0.79 | 0.1746 | 0.1655 | 0.0116 | 0.3349 | 0.3224 | 0.034 |
| **2XUTA** | **456** | 14 | 1040 | 0.71 | 0.1734 | 0.1634 | 0.0115 | 0.4442 | 0.3969 | 0.0509 |
| **3ZUXA** | **308** | 10 | 1005 | 0.9 | 0.2391 | 0.2357 | 0.0165 | 0.5395 | 0.5143 | 0.0938 |
| **2XQ2A** | **538** | 15 | 1380 | 0.82 | 0.1754 | 0.1463 | 0.009 | 0.4126 | 0.3678 | 0.0414 |
| **3M71A** | **306** | 10 | 646 | 0.97 | 0.2790 | 0.2519 | 0.019 | 0.5796 | 0.5613 | 0.1075 |
| **3QE7A** | **407** | 14 | 1355 | 0.82 | 0.1960 | 0.1754 | 0.0128 | 0.3531 | 0.3406 | 0.0392 |
| **AVERAGE** | **376** | 9.68 | 1055 | 0.875 | 0.2481 | 0.2279 | 0.0168 | 0.4845 | 0.4571 | 0.0635 |

|  | **DI Naïve L/2 ms 6** | | | **DI Processed Filt L/2 ms 6** | | | **Best DT 1L ms 12** | | | **Best ANN 3L ms 12** | | |
| --- | --- | --- | --- | --- | --- | --- | --- | --- | --- | --- | --- | --- |
| **PDBID** | **Best** | **Top 10 Avg** | **S.D.** | **Best** | **Top 10 Avg** | **S.D.** | **Best** | **Top 10 Avg** | **S.D.** | **Best** | **Top 10 Avg** | **S.D.** |
| **3NCYA** | 0.2913 | 0.2666 | 0.0259 | 0.2419 | 0.2266 | 0.0188 | 0.2847 | 0.2331 | 0.0209 | 0.2469 | 0.2275 | 0.0198 |
| **2RH1A** | 0.296 | 0.2646 | 0.0216 | 0.2998 | 0.2861 | 0.0241 | 0.3268 | 0.3107 | 0.0297 | 0.3394 | 0.3070 | 0.0271 |
| **1OKCA** | 0.3679 | 0.3287 | 0.0314 | 0.3187 | 0.3041 | 0.0307 | 0.3359 | 0.3295 | 0.0388 | 0.3489 | 0.3243 | 0.0341 |
| **1XQFA** | 0.2796 | 0.2544 | 0.0254 | 0.3118 | 0.2712 | 0.0258 | 0.2991 | 0.2745 | 0.0294 | 0.2613 | 0.2425 | 0.0221 |
| **3GD8A** | 0.4972 | 0.4785 | 0.0679 | 0.4574 | 0.4397 | 0.0448 | 0.4911 | 0.4619 | 0.0538 | 0.525 | 0.4874 | 0.0603 |
| **1L7VA** | 0.2976 | 0.2793 | 0.0251 | 0.3011 | 0.2803 | 0.0248 | 0.3132 | 0.3015 | 0.0292 | 0.3314 | 0.2976 | 0.0265 |
| **3MKTA** | 0.2551 | 0.2348 | 0.0228 | 0.2755 | 0.2511 | 0.0250 | 0.2805 | 0.2517 | 0.0253 | 0.3000 | 0.2812 | 0.0311 |
| **3RKON** | 0.255 | 0.2395 | 0.0248 | 0.2698 | 0.2487 | 0.0268 | 0.2377 | 0.2090 | 0.0176 | 0.2889 | 0.2651 | 0.0286 |
| **1OCCA** | 0.4178 | 0.3799 | 0.046 | 0.3991 | 0.3821 | 0.0488 | 0.4147 | 0.3529 | 0.0403 | 0.3655 | 0.3095 | 0.0319 |
| **1OCCC** | 0.5288 | 0.5012 | 0.0640 | 0.4944 | 0.4754 | 0.0563 | 0.4784 | 0.455 | 0.0516 | 0.4845 | 0.4702 | 0.0592 |
| **1PP9C** | 0.3796 | 0.3525 | 0.0471 | 0.3440 | 0.3208 | 0.0379 | 0.3836 | 0.3384 | 0.0392 | 0.2598 | 0.2541 | 0.0250 |
| **3H90A** | 0.4393 | 0.4139 | 0.0446 | 0.3825 | 0.3645 | 0.0347 | 0.4099 | 0.3752 | 0.0408 | 0.3977 | 0.3910 | 0.0437 |
| **3B45A** | 0.4918 | 0.4466 | 0.0498 | 0.4329 | 0.4135 | 0.0403 | 0.4779 | 0.4323 | 0.0552 | 0.4482 | 0.4336 | 0.0482 |
| **1PW4A** | 0.2793 | 0.2446 | 0.0233 | 0.3265 | 0.2583 | 0.0243 | 0.2583 | 0.2426 | 0.0228 | 0.2508 | 0.2377 | 0.0213 |
| **3DHWA** | 0.3661 | 0.3509 | 0.0277 | 0.3697 | 0.3463 | 0.0279 | 0.3485 | 0.3279 | 0.0252 | 0.3558 | 0.3322 | 0.0256 |
| **1YMGA** | 0.4422 | 0.4194 | 0.0514 | 0.4343 | 0.4098 | 0.0497 | 0.4236 | 0.4039 | 0.0514 | 0.4770 | 0.4325 | 0.0472 |
| **3B60A** | 0.2839 | 0.2647 | 0.0245 | 0.2735 | 0.2548 | 0.0236 | 0.2332 | 0.2168 | 0.0158 | 0.2585 | 0.2348 | 0.0189 |
| **2A65A** | 0.3341 | 0.3028 | 0.0353 | 0.2922 | 0.2751 | 0.0307 | 0.2708 | 0.2475 | 0.0247 | 0.2574 | 0.2389 | 0.0228 |
| **1HZXA** | 0.1992 | 0.1825 | 0.0114 | 0.1958 | 0.1836 | 0.0113 | 0.1897 | 0.1821 | 0.0108 | 0.2032 | 0.1895 | 0.0113 |
| **3PJZA** | 0.1822 | 0.1759 | 0.014 | 0.1851 | 0.1744 | 0.0137 | 0.1885 | 0.1753 | 0.0148 | 0.2097 | 0.1901 | 0.0145 |
| **2XUTA** | 0.1937 | 0.1748 | 0.0137 | 0.1756 | 0.1670 | 0.0124 | 0.234 | 0.2199 | 0.0233 | 0.2275 | 0.2125 | 0.0205 |
| **3ZUXA** | 0.3496 | 0.3315 | 0.0354 | 0.3529 | 0.3342 | 0.0352 | 0.3421 | 0.3237 | 0.0345 | 0.3745 | 0.315 | 0.0311 |
| **2XQ2A** | 0.1392 | 0.1350 | 0.0088 | 0.1555 | 0.1441 | 0.0098 | 0.1644 | 0.1417 | 0.0095 | 0.1742 | 0.1498 | 0.0099 |
| **3M71A** | 0.3514 | 0.3080 | 0.031 | 0.3675 | 0.312 | 0.0299 | 0.3498 | 0.3245 | 0.0329 | 0.3776 | 0.2948 | 0.0243 |
| **3QE7A** | 0.2484 | 0.2296 | 0.0223 | 0.2532 | 0.2291 | 0.0228 | 0.2317 | 0.2178 | 0.0192 | 0.2359 | 0.2104 | 0.0173 |
| **AVERAGE** | 0.3267 | 0.3024 | 0.0318 | 0.3164 | 0.2941 | 0.0292 | 0.3187 | 0.2940 | 0.0303 | 0.3200 | 0.2932 | 0.0289 |

TMscore results

|  | **DI Filtered MSA Stats** | | | | **Negative Best RMSD100** | | | **Pos. Control 2L ms 12** | | |
| --- | --- | --- | --- | --- | --- | --- | --- | --- | --- | --- |
| **PDBID** | ***L*** | ***TM_helix_*** | ***M_eff_*** | ***Cov*** | **Best** | **Top 10 Avg** | **S.D.** | **Best** | **Top 10 Avg** | **S.D.** |
| **3NCYA** | **422** | 12 | 1205 | 0.89 | 0.3429 | 0.3293 | 0.0252 | 0.7766 | 0.7462 | 0.1244 |
| **2RH1A** | **442** | 8 | 451 | 0.65 | 0.3645 | 0.3466 | 0.0294 | 0.613 | 0.5776 | 0.0830 |
| **1OKCA** | **292** | 6 | 1043 | 0.89 | 0.3493 | 0.3373 | 0.0262 | 0.619 | 0.6009 | 0.1013 |
| **1XQFA** | **362** | 11 | 1021 | 0.96 | 0.3873 | 0.3613 | 0.0313 | 0.7687 | 0.7618 | 0.1446 |
| **3GD8A** | **223** | 7 | 1062 | 0.96 | 0.4758 | 0.4298 | 0.0353 | 0.7175 | 0.6913 | 0.1107 |
| **1L7VA** | **324** | 10 | 1045 | 0.91 | 0.4496 | 0.3838 | 0.031 | 0.7674 | 0.7512 | 0.1338 |
| **3MKTA** | **460** | 12 | 1068 | 0.92 | 0.388 | 0.3550 | 0.0297 | 0.6974 | 0.6422 | 0.0759 |
| **3RKON** | **473** | 14 | 1722 | 0.83 | 0.3334 | 0.3251 | 0.0258 | 0.7562 | 0.7025 | 0.0937 |
| **1OCCA** | **514** | 12 | 754 | 0.98 | 0.4082 | 0.3972 | 0.0376 | 0.7553 | 0.7451 | 0.1043 |
| **1OCCC** | **261** | 6 | 521 | 0.96 | 0.4732 | 0.4463 | 0.0387 | 0.7524 | 0.7276 | 0.0657 |
| **1PP9C** | **379** | 8 | 581 | 0.92 | 0.3734 | 0.3532 | 0.0283 | 0.6875 | 0.6525 | 0.0891 |
| **3H90A** | **283** | 6 | 1039 | 0.97 | 0.4222 | 0.3866 | 0.033 | 0.6938 | 0.6539 | 0.0818 |
| **3B45A** | **180** | 6 | 1073 | 0.87 | 0.4551 | 0.4202 | 0.0361 | 0.6793 | 0.6508 | 0.0947 |
| **1PW4A** | **434** | 12 | 1604 | 0.88 | 0.3844 | 0.3609 | 0.0306 | 0.7657 | 0.7494 | 0.1076 |
| **3DHWA** | **203** | 5 | 1065 | 0.88 | 0.3684 | 0.3506 | 0.0238 | 0.5225 | 0.5104 | 0.0506 |
| **1YMGA** | **233** | 7 | 1010 | 0.9 | 0.4351 | 0.4141 | 0.0358 | 0.7083 | 0.6999 | 0.1141 |
| **3B60A** | **572** | 6 | 1568 | 0.88 | 0.3572 | 0.3314 | 0.0286 | 0.5707 | 0.5336 | 0.0557 |
| **2A65A** | **510** | 12 | 1043 | 0.83 | 0.3785 | 0.3169 | 0.0221 | 0.7974 | 0.7587 | 0.1266 |
| **1HZXA** | **340** | 7 | 1151 | 0.8 | 0.3018 | 0.2894 | 0.0232 | 0.2683 | 0.2449 | 0.0138 |
| **3PJZA** | **468** | 12 | 923 | 0.79 | 0.3386 | 0.3115 | 0.0237 | 0.5681 | 0.5526 | 0.0629 |
| **2XUTA** | **456** | 14 | 1040 | 0.71 | 0.331 | 0.3222 | 0.0269 | 0.6954 | 0.6491 | 0.0846 |
| **3ZUXA** | **308** | 10 | 1005 | 0.9 | 0.3883 | 0.383 | 0.03 | 0.7465 | 0.7355 | 0.1353 |
| **2XQ2A** | **538** | 15 | 1380 | 0.82 | 0.3432 | 0.3083 | 0.023 | 0.6958 | 0.656 | 0.0785 |
| **3M71A** | **306** | 10 | 646 | 0.97 | 0.4642 | 0.4090 | 0.0353 | 0.7879 | 0.7749 | 0.1540 |
| **3QE7A** | **407** | 14 | 1355 | 0.82 | 0.3615 | 0.3290 | 0.0275 | 0.5875 | 0.5772 | 0.0706 |
| **AVERAGE** | **376** | 9.68 | 1055 | 0.875 | 0.387 | 0.3599 | 0.0295 | 0.6799 | 0.6538 | 0.0943 |

|  | **DI Naïve L/2 ms 6** | | | **DI Processed Filt L/2 ms 6** | | | **Best DT 1L ms 12** | | | **Best ANN 3L ms 12** | | |
| --- | --- | --- | --- | --- | --- | --- | --- | --- | --- | --- | --- | --- |
| **PDBID** | **Best** | **Top 10 Avg** | **S.D.** | **Best** | **Top 10 Avg** | **S.D.** | **Best** | **Top 10 Avg** | **S.D.** | **Best** | **Top 10 Avg** | **S.D.** |
| **3NCYA** | 0.4855 | 0.4605 | 0.0476 | 0.4506 | 0.4162 | 0.0381 | 0.4783 | 0.4288 | 0.0431 | 0.4308 | 0.402 | 0.0375 |
| **2RH1A** | 0.3902 | 0.3548 | 0.0340 | 0.3998 | 0.3872 | 0.0365 | 0.425 | 0.4113 | 0.0434 | 0.4539 | 0.4139 | 0.0402 |
| **1OKCA** | 0.4999 | 0.4590 | 0.0469 | 0.4515 | 0.4307 | 0.0461 | 0.4706 | 0.4534 | 0.0560 | 0.4711 | 0.4528 | 0.0515 |
| **1XQFA** | 0.4493 | 0.4269 | 0.0433 | 0.5075 | 0.4538 | 0.0437 | 0.4622 | 0.4444 | 0.0487 | 0.4475 | 0.4070 | 0.0378 |
| **3GD8A** | 0.6534 | 0.6248 | 0.0959 | 0.5905 | 0.5767 | 0.0648 | 0.6351 | 0.6039 | 0.0772 | 0.6865 | 0.6336 | 0.0848 |
| **1L7VA** | 0.4882 | 0.4418 | 0.0436 | 0.4747 | 0.4484 | 0.0421 | 0.4752 | 0.4601 | 0.0471 | 0.5178 | 0.4657 | 0.0417 |
| **3MKTA** | 0.4631 | 0.4493 | 0.0474 | 0.5217 | 0.4923 | 0.0531 | 0.5291 | 0.4716 | 0.0492 | 0.5622 | 0.532 | 0.0609 |
| **3RKON** | 0.4584 | 0.4449 | 0.0472 | 0.5077 | 0.4666 | 0.0509 | 0.4253 | 0.3944 | 0.0360 | 0.5551 | 0.4918 | 0.0538 |
| **1OCCA** | 0.6746 | 0.6362 | 0.0805 | 0.6386 | 0.6308 | 0.0851 | 0.6772 | 0.6066 | 0.0732 | 0.6143 | 0.5431 | 0.0620 |
| **01OCCC** | 0.6496 | 0.6244 | 0.0826 | 0.6145 | 0.5865 | 0.0717 | 0.6144 | 0.5750 | 0.0698 | 0.6205 | 0.5852 | 0.0733 |
| **1PP9C** | 0.539 | 0.5178 | 0.0679 | 0.5221 | 0.4845 | 0.0567 | 0.5611 | 0.5106 | 0.0579 | 0.4149 | 0.3971 | 0.0366 |
| **3H90A** | 0.5549 | 0.5168 | 0.0611 | 0.4668 | 0.4536 | 0.0488 | 0.4811 | 0.4648 | 0.0557 | 0.5134 | 0.4935 | 0.0600 |
| **3B45A** | 0.6005 | 0.5464 | 0.0650 | 0.5312 | 0.5122 | 0.052 | 0.5853 | 0.5432 | 0.0743 | 0.5706 | 0.54 | 0.0629 |
| **1PW4A** | 0.5059 | 0.4649 | 0.0506 | 0.5752 | 0.4976 | 0.0532 | 0.5042 | 0.4609 | 0.0489 | 0.4621 | 0.4494 | 0.0443 |
| **3DHWA** | 0.4516 | 0.4369 | 0.0356 | 0.4514 | 0.432 | 0.0345 | 0.4249 | 0.4153 | 0.0319 | 0.4532 | 0.4228 | 0.0318 |
| **1YMGA** | 0.5856 | 0.5654 | 0.0765 | 0.5973 | 0.5578 | 0.0758 | 0.5749 | 0.5530 | 0.0775 | 0.6128 | 0.5747 | 0.0673 |
| **3B60A** | 0.3831 | 0.3591 | 0.0357 | 0.3716 | 0.3557 | 0.0346 | 0.3044 | 0.2934 | 0.025 | 0.3539 | 0.3319 | 0.0294 |
| **2A65A** | 0.5699 | 0.5365 | 0.0616 | 0.5554 | 0.507 | 0.0575 | 0.4985 | 0.4499 | 0.0465 | 0.4876 | 0.4565 | 0.0454 |
| **1HZXA** | 0.2638 | 0.2438 | 0.0155 | 0.2626 | 0.2518 | 0.0167 | 0.2587 | 0.2529 | 0.017 | 0.2835 | 0.2645 | 0.0178 |
| **3PJZA** | 0.3529 | 0.3224 | 0.0290 | 0.3503 | 0.3259 | 0.0290 | 0.3521 | 0.3380 | 0.0322 | 0.3877 | 0.3626 | 0.0333 |
| **2XUTA** | 0.3339 | 0.3260 | 0.0275 | 0.3323 | 0.3186 | 0.0264 | 0.4214 | 0.3993 | 0.0429 | 0.4255 | 0.3992 | 0.0385 |
| **3ZUXA** | 0.5476 | 0.5203 | 0.0560 | 0.5375 | 0.5231 | 0.0566 | 0.5132 | 0.5030 | 0.0528 | 0.5776 | 0.4980 | 0.0512 |
| **2XQ2A** | 0.3172 | 0.2994 | 0.0247 | 0.3338 | 0.3129 | 0.0258 | 0.3307 | 0.3080 | 0.0259 | 0.3371 | 0.3206 | 0.0260 |
| **3M71A** | 0.5328 | 0.4864 | 0.0530 | 0.5792 | 0.4862 | 0.0509 | 0.5424 | 0.4997 | 0.056 | 0.5307 | 0.4653 | 0.0439 |
| **3QE7A** | 0.438 | 0.4155 | 0.0422 | 0.4513 | 0.4083 | 0.0414 | 0.4192 | 0.3975 | 0.0376 | 0.4322 | 0.3838 | 0.0338 |
| **AVERAGE** | 0.4876 | 0.4592 | 0.0508 | 0.483 | 0.4527 | 0.0477 | 0.4786 | 0.4496 | 0.049 | 0.4881 | 0.4515 | 0.0466 |

Here we have replicated Table 3 of main manuscript containing the same results, but with GDT-TS and TMscore as similarity metrics. While not as wide-spread as TM-score or GDT-TS, RMSD100 is also a metric for length-independent comparison of structural similarity and enables comparison to prior work using BCL::Fold.
